# Phenotype-driven gene prioritization for rare diseases using graph convolution on heterogeneous networks

**DOI:** 10.1186/s12920-018-0372-8

**Published:** 2018-07-06

**Authors:** Aditya Rao, Saipradeep VG, Thomas Joseph, Sujatha Kotte, Naveen Sivadasan, Rajgopal Srinivasan

**Affiliations:** TCS Research and Innovation, Hyderabad, 500081 India

**Keywords:** Rare diseases, Gene prioritization, Heterogeneous networks, Graph convolution

## Abstract

**Background:**

One of the major goals of genomic medicine is the identification of causal genomic variants in a patient and their relation to the observed clinical phenotypes. Prioritizing the genomic variants by considering only the genotype information usually identifies a few hundred potential variants. Narrowing it down further to find the causal disease genes and relating them to the observed clinical phenotypes remains a significant challenge, especially for rare diseases.

**Methods:**

We propose a phenotype-driven gene prioritization approach using heterogeneous networks in the context of rare diseases. Towards this, we first built a heterogeneous network consisting of ontological associations as well as curated associations involving genes, diseases, phenotypes and pathways from multiple sources. Motivated by the recent progress in spectral graph convolutions, we developed a graph convolution based technique to infer new phenotype-gene associations from this initial set of associations. We included these inferred associations in the initial network and termed this integrated network HANRD (Heterogeneous Association Network for Rare Diseases). We validated this approach on 230 recently published rare disease clinical cases using the case phenotypes as input.

**Results:**

When HANRD was queried with the case phenotypes as input, the causal genes were captured within Top-50 for more than 31% of the cases and within Top-200 for more than 56% of the cases. The results showed improved performance when compared to other state-of-the-art tools.

**Conclusions:**

In this study, we showed that the heterogeneous network HANRD, consisting of curated, ontological and inferred associations, helped improve causal gene identification in rare diseases. HANRD allows future enhancements by supporting incorporation of new entity types and additional information sources.

**Electronic supplementary material:**

The online version of this article (10.1186/s12920-018-0372-8) contains supplementary material, which is available to authorized users.

## Background

The success of genomic medicine is crucially dependent on rapid, comprehensive and accurate assessment of a patient’s genomic variants and the relation of these variants with the observed clinical phenotypes. Variant prioritization identifies a few hundred variants by considering the genotype. Narrowing the variant list further down to find the genes harboring these variants that are responsible for the observed clinical phenotypes remains a significant challenge [1]. This is particularly challenging in the context of rare Mendelian genetic diseases. Availability of comprehensive and precise phenotypic data of the patient can significantly aid in solving this problem [2].

One of the major goals of computational deep phenotyping [3] is to aid the analysis of genomic data for personalized genomic medicine [2]. Existing tools for this include Phenomizer [4], Phenolyzer [5] and PCAN [6], amongst others. There also exist composite gene and variant prioritization tools that combine phenotype analysis and variant analysis identified by whole exome sequencing (WES) or whole genome sequencing (WGS) for the study of human disease. These include OMIM Explorer [7], VarElect [1], Exomiser[8], OVA [9], Phevor [10], Phen-Gen [11], eXtasy [12] and the Phenotype-Driven Ranking (PDR) algorithm in Ingenuity Variant Analysis [13]. Smedley and Robinson [2] have reviewed many of these tools. These tools often require as input a set of genes known as ’seed genes’ that are already known to be associated with specific phenotypes [9]. This is a major limitation when dealing with novel associations between phenotypes and genes. Tools that can infer phenotype-genotype associations when presented with a set of input phenotypes are better placed to overcome this limitation.

Phenomizer relies on a semantic network between phenotypic terms to find potential candidate diseases and corresponding genes when presented with a set of input phenotypes. Similar network-based approaches such as GeneMANIA [14] and GUILD [15] require (1) a network of known associations between various biological entities such as genes and phenotypes, and (2) an algorithm for inferring and scoring associations using the underlying network. The associations could be ontological associations, biological interactions, or ‘associations by guilt’ where the participating entities co-occur in some context [16]. Algorithms for inferring and scoring associations include CIPHER [17], PRINCE [18], Random walk with restart on heterogeneous network (RWRH) [19], Bi-Random Walk (BiRW) [20] and MAXimum Information Flow (MAXIF) [21]. CIPHER connects protein interaction networks and the phenotype network to try and predict disease genes. PRINCE uses label propagation on networks for association scoring. The RWRH algorithm, when applied to gene prioritization, ranks genes and phenotypes simultaneously in a network built using phenotype-gene associations from the Online Mendelian Inheritance in Man (OMIM) catalog. BiRW computes novel phenotype-gene associations by exploring special sub-graph structures called circular bigraphs in the underlying network. A circular bigraph is defined in [20] as consisting of a phenotype only path and a gene only path whose endpoints are connected by phenotype-gene links. These structures capture the biological intuition that a new phenotype-gene link would ideally be present in the current network as a path comprising of a phenotype subpath followed by a gene subpath. The phenotype subpath captures the onotological relations and gene subpath captures a sequence of known gene-gene associations. MAXIF uses network flow for association scoring. BiRW has been shown to outperform other network-based algorithms such as PRINCE, CIPHER and RWRH [20].

Identifying causal genes that best explain a set of clinical phenotypes using network-based prioritization approaches remains a challenging task [22], especially for rare diseases. We describe the construction of a heterogeneous network consisting of entities such as genes, phenotypes, diseases and pathways as nodes while associations between these entities are represented as weighted edges. The weight of an edge represents the score of the association between the entity pairs. Existing association networks usually view ontological associations as distinct from the network of other heterogeneous associations [22]. We instead combine pairwise ontological and curated associations into a single heterogeneous association network. Motivated by the recent progress in spectral graph convolutions [23, 24], we develop an information propagation algorithm GCAS (*Graph Convolution-based Association Scoring*) that performs information propagation on the initial ontological and curated association network and infers novel binary associations between the entities of the network. These inferred associations are added to the aforementioned initial network, and the resulting network of ontological, curated and inferred associations is called HANRD for *Heterogeneous Association Network for Rare Diseases*. We built HANRD to solve the specific problem of phenotype-driven rare disease gene prioritization wherein the input is a set of phenotypes from clinical cases and the output a ranked list of possible causal genes.

## Methods

In this section, we describe the construction of an initial heterogeneous network consisting of ontological and curated associations. We then describe in detail our association inference algorithm GCAS. GCAS is applied to the initial network to obtain inferred associations, which are added to the initial ontological and curated associations to create HANRD. A series of comparisons are then performed to analyze the performance of GCAS and HANRD. We validated the use of HANRD for gene prioritization on a dataset of 230 solved rare disease clinical cases reported in recent publications. The performance of HANRD on these cases was compared to that of Phenomizer (Orphanet) and BiRW. We also examined the impact of the inferred associations by excluding them from HANRD i.e., performing gene prioritization for the 230 cases on the initial network of ontological and curated associations. The performance of GCAS with an adaptation of GCN (*Graph Convolutional Network*) [24] was also compared using the 230 solved clinical cases.

### HANRD network construction

Entities of type disease, phenotype, gene and pathway are the nodes of our heterogeneous network HANRD. Human Phenotype Ontology (HPO) [3] was the primary source for phenotypes. HPO names and synonyms were augmented with additional synonyms from the Medical Subject Headings (MeSH) resource via cross-references provided by HPO and HPO-UMLS mappings [25]. Orphanet was used as the primary source for diseases. Each Orphanet record contains the rare disease name, synonyms, descriptions, associated phenotypes (including association strength), associated genes as well as MeSH mappings. Additional disease synonyms were obtained through MeSH mappings, wherever provided by Orphanet. Gene names were derived from the HUGO Gene Nomenclature Committee (HGNC) database [26] (accessed 15th Feb, 2017). Names of biological pathways were extracted from Wiki Pathways [27, 28]. The final set of phenotype, disease, gene and pathway terms were used as nodes in HANRD. The main term was propagated as the node label, while other terms were propagated as node synonyms.

**Fig. 1:**
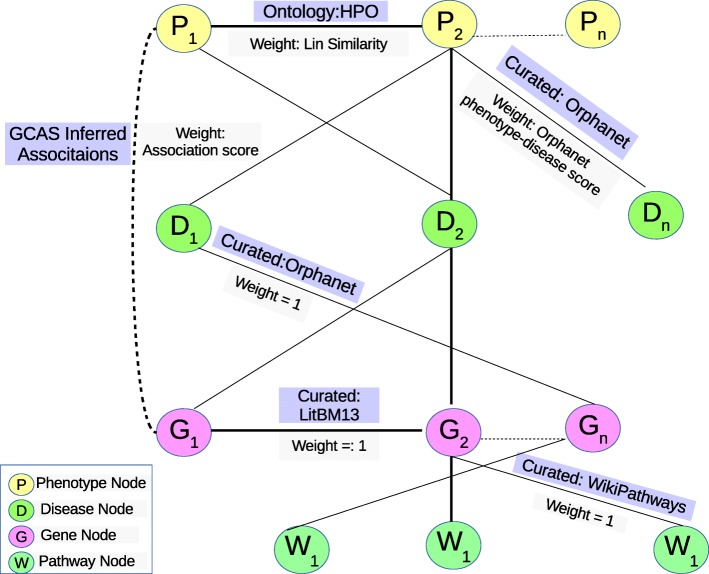
The figure shows the various curated, ontological and inferred association types in HANRD. The HANRD edges could be between Phenotypes, Diseases, Genes and Pathways. The edges are undirected and weighted. The dotted lines indicate the inferred edges. Along with each edge type, the weight assignment scheme and the information source for the edge is also shown

The associations between the above nodes are represented by undirected edges with non-negative edge weights. The first set of associations incorporated were the phenotype-phenotype edges constructed from the HPO ontology. Weights for phenotype-phenotype edges were calculated using the standard Lin similarity score for ontological associations [29]. The Lin similarity score *s*(*p*_1_,*p*_2_) between two phenotypes *p*_1_ and *p*_2_ is given by 2*IC*(*p*^′^)/(*IC*(*p*_1_)+*IC*(*p*_2_)), where *p*^′^ is the most specific common ancestor of *p*_1_ and *p*_2_ in the ontology hierarchy while *IC*(*p*) is the information content of phenotype *p*. We used *IC*(*p*)=− ln(*f*(*p*)/*N*)) as in HPOSim [29], where *f*(*p*) is the frequency of *p* and its descendants in a corpus and *N* the total frequency. Disease data from Orphanet was used to build phenotype-disease edges, with the frequency qualifiers from Orphanet used to calculate the edge weights. The frequency qualifiers include terms such as “obligate”, “very frequent” and “frequent”. Orphanet data was also used as the source for disease-gene pairs, with the edge weights for these pairs were set to 1. A high-quality curated interaction dataset called Lit-BM-13 (downloaded on January 11th, 2017) was the source of curated gene-gene associations and the corresponding edges were assigned weight 1 [30]. Wiki Pathways was used for pathway associations, wherein every gene present in a pathway was linked to the corresponding pathway node with a separate edge having weight 1 [27, 28]. Figure 1 shows the various curated and ontological association types in HANRD.

The network of curated associations was augmented with a set of inferred associations obtained by performing GCAS on this initial network. The motivation behind using GCAS was to use graph convolution to propagate information between entity pairs in a network and use this propagated information to determine association scores between entity pairs having no direct links. For GCAS, the initial curated network is assumed to be static and given as input. The mathematical description of GCAS is as follows:

Propagation of a signal $x \in \mathbb {R}^{n}$ on a given network *G* consisting of *n* nodes can be viewed as the convolution of *x* with a filter *g* on the network *G*. Let *A*_*n*×*n*_ be the adjacency matrix of *G* and *L* be the normalized graph Laplacian of *G* given by $L = I_{n} - D^{-\frac {1}{2}} A D^{-\frac {1}{2}} = U \Lambda U^{T}$ where *I*_*n*_ is the identity matrix, *D* is the diagonal degree matrix with $D_{i i} = \sum _{j} A_{i j}$, *U* is the matrix of eigenvectors of *L* and *Λ* is the diagonal matrix of eigenvalues of *L*. By [23], spectral convolution of *x* with the filter *g* on the network *G* can be equivalently represented as *g*_*Θ*_⋆*x*=*Ug*_*Θ*_ *U*^*T*^*x*, where *U*^*T*^*x* is the graph Fourier transform of *x* and *g*_*Θ*_=*diag*(*Θ*) is a diagonal matrix corresponding to $\Theta \in \mathbb {R}^{n}$, which is the graph Fourier transform of the filter *g*. Here, *g*_*Θ*_ *U*^*T*^*x* gives the pointwise multiplication of the Fourier transforms of *g* and *x*. Multiplication of *U* with *g*_*Θ*_ *U*^*T*^*x* in *g*_*Θ*_⋆*x* gives the Fourier inverse. We refer the reader to [23] for a detailed treatment on graph Fourier transforms. Our aim is to design the filter *g*_*Θ*_ that achieves the desired signal propagation on *G*.

To handle the computational overhead and numerical instabilities, a first order approximation of *g*_*Θ*_⋆*x* based on the Chebyshev polynomial approximation of *g*_*Θ*_ has been used [23, 24]. Under this approximation, $g_{\Theta } \star x \approx \theta \hat {A} x$, where $\theta \in \mathbb {R}$ is a single parameter, $\hat {A} = \tilde {D}^{-\frac {1}{2}} \tilde {A} \tilde {D}^{-\frac {1}{2}}$, $\tilde {A} = A + I_{n}$ and $\tilde {D}$ is a diagonal matrix with $\tilde {D}_{ii} = \sum _{j} \tilde {A}_{ij}$. Our algorithm for inferring associations uses this approximation of the convolution operation and computes the information propagated to the *t*th order neighborhood of the network nodes by performing *t* consecutive applications of this convolution operation. After *t* consecutive convolutions, the resulting values at the network nodes is given by the vector $C_{t}(x) = {\theta ^{\prime }} \hat {A}^{t} x$ where ${\theta ^{\prime }} = \prod _{i=1}^{t} \theta _{i}$ and *θ*_*i*_ is the parameter for the *i*th convolution. We use this information propagation model and fix *θ*_*i*_=*θ* for all *i*>2 where *θ* is a parameter and use another parameter *K* which bounds the convolution depth to compute the final pairwise association score matrix *S* as:

$$ S = \sum\limits_{t=1}^{K} C_{t}(I_{n}) $$

where $C_{t}(I_{n}) = \theta ^{t-2} \hat {A}^{t} I_{n}$ for *t*≥2 and $C_{1}(I_{n}) = \hat {A} I_{n}$. We consider only the off-diagonal entries of matrix *S*. The key parameters of the algorithm are thus *K* and *θ*. Parameter *K* restricts the inferred associations to entity pairs that are at most *K* links away in the network. The parameter *θ*∈ [0,1] can be understood as the damping or penalizing factor that dampens information flow along longer paths. The damping increases by a multiplicative *θ* for every additional link in path. By choosing *θ*^*t*−2^ as the parameter in *C*_*t*_(*I*_*n*_), the damping is applied only for information flow along paths having three or more links. We refer to our inference algorithm as GCAS, for *Graph Convolution-based Association Scoring*.

The values for parameters *K* and *θ* were selected by performing a grid search across a range of values. For each combination of *K* and *θ* values, GCAS was run on multiple random sub-networks of the original network and its performance for inferring missing associations in the sub-network was analyzed. The final parameter values chosen were *K*=9 and *θ*=0.25.

Inferred associations were obtained by running GCAS on the initial curated network with these parameters. These inferred associations together with the ontological and curated associations form the heterogeneous network HANRD. Figure 1 shows the various curated and inferred association types present in HANRD. HANRD is used for the gene prioritization task as follows. Given a set of input disease phenotypes, their gene neighbors in HANRD were ranked based on their cumulative association score with respect to the input phenotypes, where the cumulative score is given by the sum of the association scores with individual phenotypes.

As stated earlier, the first-order approximation of the spectral convolution from [24] was used in our information propagation model. In [24], a GCN (*Graph Convolutional Network*), which is a convolutional neural network based on spectral graph convolution, was proposed for semi-supervised node classification in graphs. Each layer of the GCN neural network is based on the same first order approximation of the spectral graph convolution together with point-wise non-linearity. Two-layer GCN was used for node classification tasks in citation and knowledge networks in [24]. GCAS shares resemblance to GCN in the sense that both approaches are based on spectral graph convolution [23]. However, the cross-entropy based error model in GCN makes it more suitable for inferring the cumulative association of a sufficiently large set of related nodes in the graph to the remaining nodes rather than inferring individual pair-wise associations. Furthermore, realizing convolution with the *K*^*th*^ order neighborhood require deeper networks in GCN which leads to increase in the number of parameters and could also lead to overfitting [24]. On the other hand, GCAS performs direct spectral convolution (using the first order approximation) successively with the chosen filter parameters to efficiently propagate the signal to the *K*^*th*^ order neighborhood of a node. This allows efficient estimation of long range associations from each single node to its *K*^*th*^ order neighborhood for large *K* values.

The BiRW algorithm has previously been shown to outperform other state-of-the-art network inference algorithms [20]. We conducted an experiment to compare the performance of BiRW and GCAS for inferring novel associations. In the original BiRW implementation [20], the nodes represent genes and disease phenotypes, while the edges are phenotype-gene associations from OMIM [31] as well as protein-protein interactions (PPI) and phenotype-phenotype associations. In order to perform the experiment, we constructed an instance of HANRD called HANRD _trunc_ consisting of only phenotype and gene nodes and only the curated associations involving them. This was done by removing all intermediate disease and pathway nodes in HANRD and introducing direct connections between the genes and phenotypes nodes. Parameters for BiRW were assigned the same optimal values as in the original implementation [20]. BiRW requires OMIM disease phenotypes as input, using the corresponding OMIM IDs. On the other hand, GCAS has been designed to take HPO phenotypes (HPO IDs) as input. Hence, the BiRW implementation was modified to handle HPO IDs as input. The modified implementation is referred to as BiRW _mod_.

For comparison, we performed 10-fold cross validation by running both GCAS and BiRW _mod_ on HANRD _trunc_. In each fold, 10% (670) phenotype-gene links were removed from HANRD _trunc_ at random. Both methods were run on the remaining network to augment it with inferred associations. The removed phenotype-gene associations were used as test data. The AUC _*N*_ (Area Under the Curve) value of the ROC (Receiver Operating Characteristic) curve [20, 32] was computed separately for each test phenotype. The ROC score was derived based on the ranks of the target genes associated with the phenotype among all its gene neighbors in the network. For AUC _*N*_, the number of false positives are limited to be at most *N* [20]. The average AUC _*N*_ value was computed within a fold. Although removal of several existing network edges can affect the overall performance and thereby results in lower AUC values, these values can nevertheless be used for comparison of the two algorithms.

### Validation on rare disease clinical data

We built HANRD to solve the specific task of phenotype-driven rare disease gene prioritization wherein the input is a set of phenotypes from clinical cases and the output a ranked list of possible causal genes. We validated its application to this task using a dataset of solved rare disease clinical cases reported in recent publications [1, 33–35]. This dataset, included in the Additional file 1, had a list of clinical phenotype terms for each case along with the diagnosed disease(s) and the corresponding causal gene(s). The clinical cases from Stavropoulos et al. [33] enforced HPO coding of the phenotypic terms using the Phenotips tool [36]. Since we did not have access to this HPO coding, we manually assigned HPO codes for the clinical phenotypic terms using a verbatim search via the HPO browser interface. For cases from the remaining sources, we manually analyzed each phenotypic term and assigned HPO codes. Any disease term mentioned in the phenotype description was ignored. These HPO IDs representing the clinical phenotype of a case served as the input query to HANRD. For each HPO ID, HANRD was queried resulting in a ranked list of genes. After iterating over all input HPOs, a single list of ranked genes was obtained. We checked for the rank of the known causal gene, if present, in this list.

We compared the performance of using HANRD for the 230 solved cases with that of Orphamizer. Since HANRD uses Orphanet data, Orphamizer was chosen instead of Phenomizer. Here, each case consists of a set of clinical phenotype terms represented by HPO IDs while the corresponding output was a ranked list of associated genes. We considered the cumulative distribution of the number of input phenotype-genotype pairs for different Top-*k* values. For different Top-*k* values, the percentage of phenotype-gene pairs where the gene appeared within the Top-*k* of the ranked list of genes for the phenotype was measured.

We also compared GCAS with BiRW in the specific context of these 230 real-world cases. Both GCAS and the modified BiRW (BiRW _mod_) were run on HANRD _trunc_ for these cases. For each of the 230 cases, we built an association pair for each phenotype and causal gene(s). Since BiRW _mod_ produces a separate ranked list of genes for each phenotype term of the input phenotype list, each input phenotype was analyzed separately.

Further, we evaluated the GCN implementation [24, 37] on these 230 cases. The weighted graph constructed from the initial curated and ontological associations formed the input graph. GCN supports capturing the known associations between entities using links in the network and also using additional node level feature similarities. In our case, the entity associations are already captured in the initial network using link structures together with link weights. However, no node level features were present in the initial network. GCN is designed for node label propagation under semi-supervised setting. For each designated clinical test case, the associated phenotypes were all assigned the same label and this formed the labeled input. GCN performed label propagation to other nodes based on the cross-entropy error for the labeled input. The final labeled scores for the genes were used for gene ranking. We experimented with GCN under two different settings for each designated clinical test case. In one setting, only the phenotypes for the designated clinical test case formed the labeled class. In the second setting, each of the remaining 229 cases were also labeled with separate labels and this labeled data was additionally provided as support. Each of these additional labeled classes consisted of both the case specific phenotypes and the causal genes. The final gene ranking was based on their corresponding scores with respect to the label associated with the designated clinical test case.

In another experiment, we measured the effect of the convolution depth parameter *K* on the performance of HANRD. Larger *K* values result in convolution with higher order neighborhood of the network nodes. We ran GCAS on a range of *K* values. In each case, the input network was augmented with the inferred associations to create a *K* value specific HANRD instance. The resulting HANRD instances were then used to solve the 230 clinical cases discussed above. For each HANRD instance, we measured the cumulative distribution of the total number causal genes found in the Top-*k* of the ranked gene lists from all the 230 cases.

## Results

### Comparison with BiRW algorithm

**Fig. 2:**
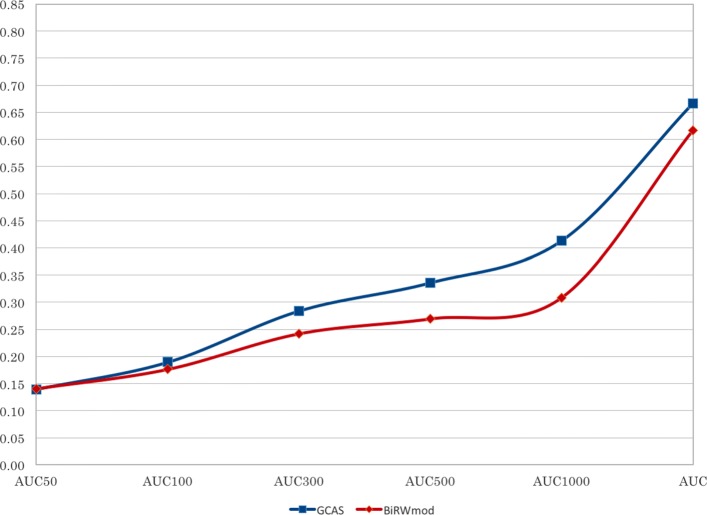
Average AUC _*N*_ with N = 50, 100, 300, 500, 1000 and the full AUC value for the 10-fold cross validation of GCAS and BiRW _mod_

Figure 2 shows the comparison of GCAS with BiRW _mod_, based on the 10-fold cross validation described in “HANRD network construction” section. The AUC _*N*_ values and the full AUC value averaged over all folds are shown here for both BiRW _mod_ and GCAS. Fold-wise values along with mean and standard deviation are given in the Additional file 2. A candidate implementation for AUC computation is available at [38]. The plot shows marginally improved performance of GCAS over BiRW _mod_ for larger Top-*k*. BiRW explores domain specific short range connections in a network involving only genes and phenotypes. GCAS on the other hand explores both long range and short range connections in a domain-independent fashion. We note that AUC values are shown only to compare the performance of the two algorithms and not to quantify the performance of any one algorithm in isolation.

### Validation on rare disease clinical data

**Fig. 3:**
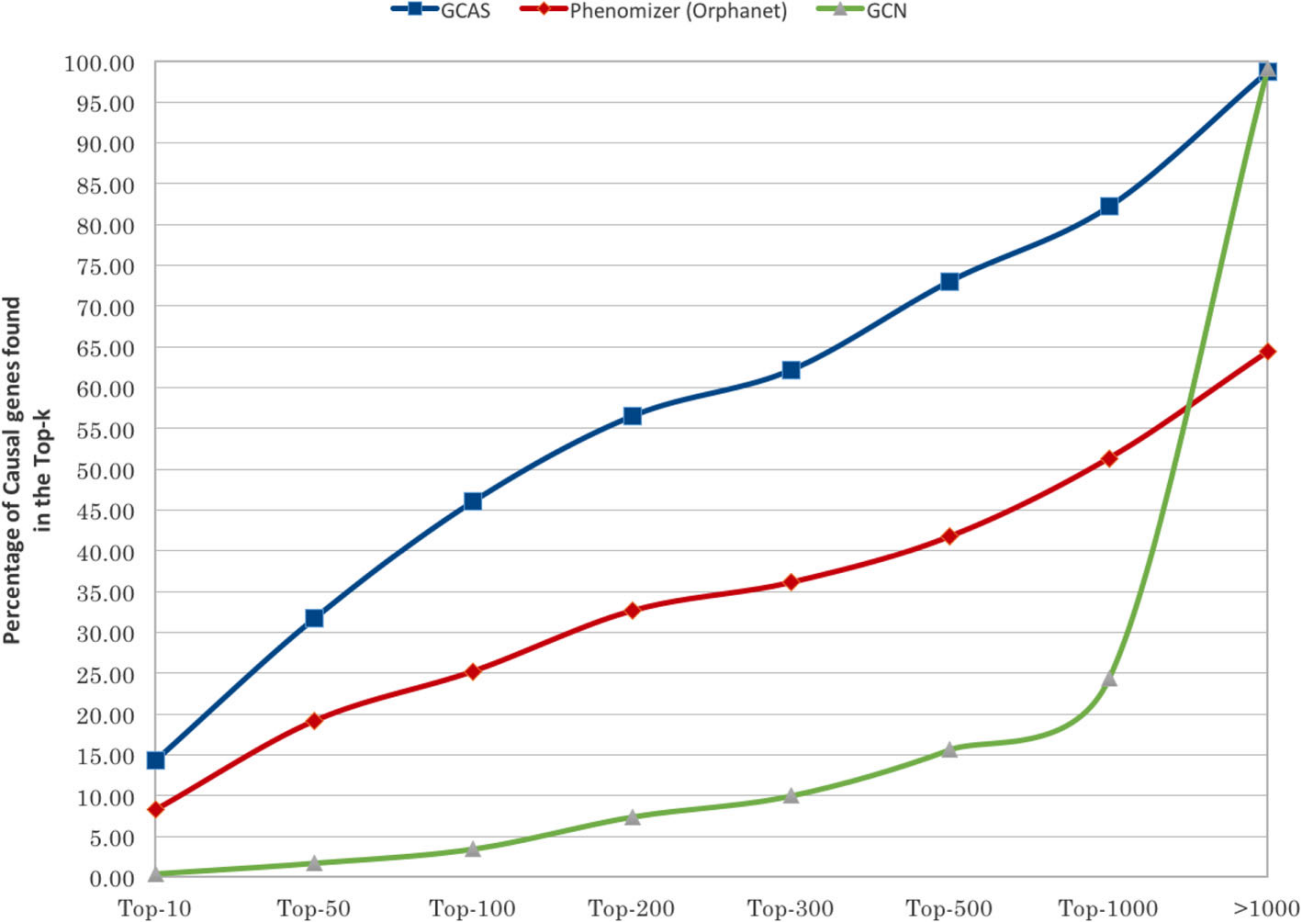
Cumulative percentage of the 230 clinical cases where the causal gene(s) appeared within the Top-*k* of the ranked list of genes. The candidate methods are GCAS, Phenomizer(Orphanet) and GCN

Figure 3 show the comparison of HANRD and Orphamizer. For different Top-*k* values, the percentage of cases where the causal gene(s) appeared within the Top-*k* of the ranked list of genes for the input set of phenotypes was plotted. The Orphamizer output was ranked disease-wise, wherein a gene could occur in the list for more than one input phenotype associated with the disease. In such cases, we assign the highest rank for the causal genes. As seen in the figure, HANRD could capture causal gene(s) for more than 31% of the cases in Top-50 and more than 56% of the cases in Top-200, when compared with Orphamizer which got 19 and 32% respectively.

**Fig. 4:**
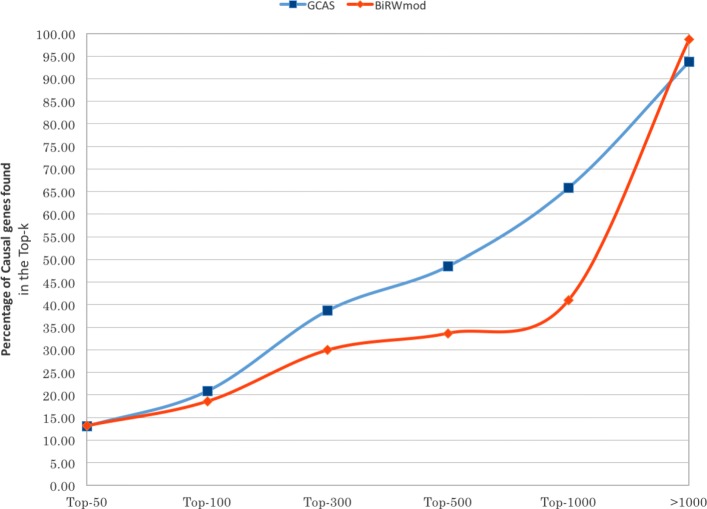
Cumulative percentage of all the phenotype-gene associations from the 230 clinical cases that appeared within the Top-*k* of the ranked gene list. The candidate methods are GCAS and BiRW _mod_

**Fig. 5:**
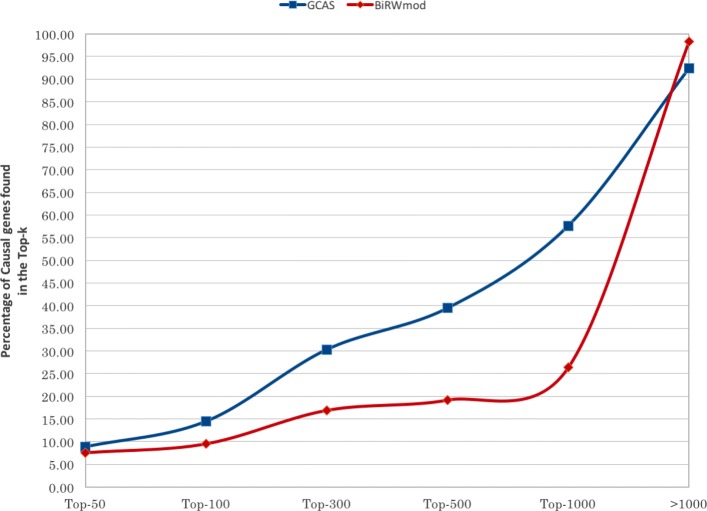
Cumulative percentage of all the phenotype-gene associations from the 230 clinical cases that appeared within the Top-*k* of the ranked gene list. The phenotype-gene associations that are already present in Orphanet are excluded from the calculation. The candidate methods are GCAS and BiRW _mod_

Figure 4 shows the comparison of GCAS and BiRW _mod_ for all the phenotype-gene pairs derived from the clinical cases. It shows the cumulative distribution of the number of phenotype-gene pairs whose genes appear in the Top-*k* of the ranked list of its phenotype. Figure 5 shows the distribution after excluding from the Top-*k* calculation those phenotype-gene pairs that are already linked in HANRD _trunc_ with non-zero association scores. This was done to avoid any performance bias due to the overlap between input pairs and inferred associations. Figures 4 and 5 show that GCAS performs considerably better than BiRW _mod_ in both evaluations. As shown in Fig. 4, BiRW and GCAS exhibit similar performance at *k*=50 while GCAS outperforms BiRW after *k*=50. In other words, GCAS identifies the causal genes for more cases if we allow a larger Top-*k*. However, as seen in Fig. 5, GCAS outperforms BiRW even at *k*=50 after excluding the phenotype-gene pairs present in HANRD _trunc_.

**Fig. 6:**
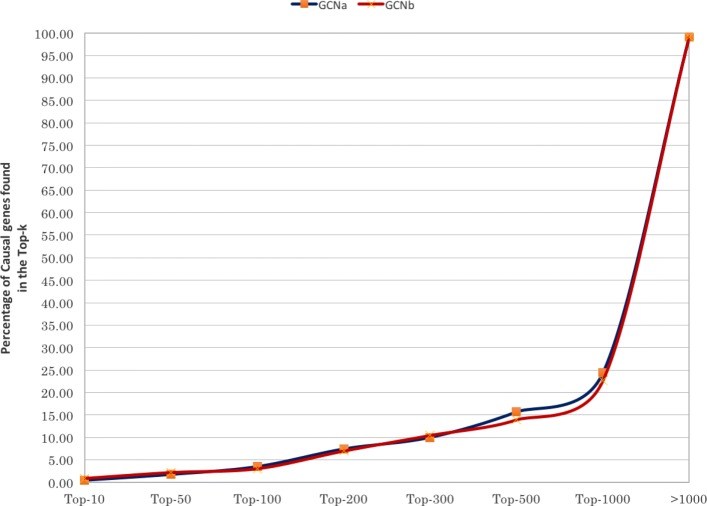
Cumulative percentage of the 230 clinical cases where the causal gene(s) appeared within the Top-*k* of the ranked list of genes. The candidate methods are GCN _*a*_ and GCN _*b*_

Figure 6 compares the GCN performance under the two settings discussed in “Validation on rare disease clinical data” section. GCN performs slightly better in the setting where only the test case is provided as labeled data (GCN _*a*_) in comparison to the setting where additional labeled information related to the remaining clinical cases (GCN _*b*_) was also provided as support. The GCN performance is not improved by the additional support data. On the contrary, the performance degradation of GCN _*b*_ could possibly be attributed to the large number of labels that are simultaneously considered while computing the final label propagation. Hence, only GCN _*a*_ is used in the comparison given in Fig. 4. As seen in Fig. 4, GCAS consistently outperforms GCN. For instance, GCN could capture only 15% in the Top-500 while Orphamizer and GCAS could capture 42 and 73% of the causal genes respectively.

**Fig. 7:**
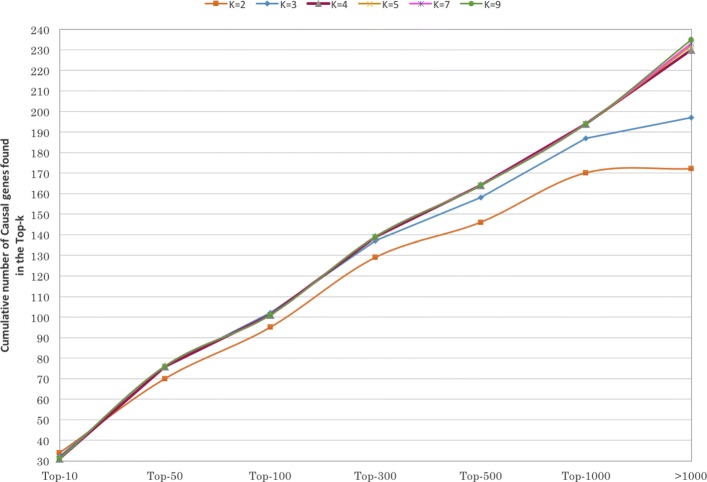
Performance of HANRD for convolution depth parameter *K*=2,3,4,5,7 and 9. For each *K*, the cumulative distribution of the number of causal genes appearing within the Top-*k* of the ranked gene lists from all the 230 clinical cases is plotted

Figure 7 show the performance of HANRD in solving the 230 clinical cases for different values of the convolutional depth parameter *K*. As discussed in “Validation on rare disease clinical data” section, a separate HANRD instance was created for each candidate *K* value. Performance of each of these HANRD instances was plotted separately as follows. For different Top-*k* values, the total number of causal genes that appeared within the Top-*k* of the ranked gene lists from all the clinical cases was plotted. GCAS showed improved performance with increasing *K* values though the improvement was marginal for *K* greater than 4.

## Discussion

We presented a phenotype-driven approach for rare disease gene prioritization consisting of a heterogeneous network HANRD as well as a spectral graph convolution algorithm GCAS for inferring pairwise associations. HANRD was built using ontological and curated associations supplemented by inferred associations. Validation on rare disease clinical cases showed improved performance of our approach when compared to other state-of-the-art tools. When the phenotypes associated with the rare disease clinical cases were presented as input, the causal genes were captured within Top-50 for more than 31% of the cases and within Top-200 for more than 56% of the cases.

GCAS showed a superior Top-*k* recall than BiRW for the rare disease clinical cases while also achieving comparable AUC scores in the cross-validation. The recall performance of GCAS and BiRW are similar for small Top-*k* (Top-50). BiRW relies on a rigid network structure and it explores short range connections between entities. As a result, BiRW exhibits comparable precision (smaller Top-*k*) for a small subset of clinical cases. On the other hand, exploring only rigid structures with short range connections result in lower recall for BiRW for most other cases. Since GCAS explores both short-range and long-range connections, it is able to achieve a better balance of precision and recall. Computing long-range associations suffers from noise since the neighborhood expands considerably for increasing *k*. As a result, causal genes may appear only in a larger Top-*k* range. Nevertheless, good recall with reasonably large Top-*k* can still significantly help in identifying causal genes in rare disease clinical cases. This is especially true when the ranked gene list output is combined with other similar lists arising from say genotyping (WES or WGS) experiments. Though the candidate genes could have lower rank in a list in isolation, combining its support from all ranked lists can produce a list of significantly higher quality than any of the individual lists and thereby help in efficient identification of the causal gene(s).

BiRW approach explores domain specific and rigid sub-structures (circular bigraphs consisting of only genes and phenotypes) in the network for inferring novel associations. GCAS on the other hand uses a domain-independent approach and it explores both short range and long range connections to infer novel associations. This makes GCAS better suited for adapting to other domains.

GCAS and Orphamizer performed better than GCN in the clinical case validation. The number of input phenotypes associated with a clinical case is usually very low (in the range of 3 to 5). GCN is perhaps not well suited for such cases requiring information propagation from a very small set of source nodes to the remaining nodes. Due to this limitation, GCN has limited applicability in augmenting a network with new inferred pair-wise associations, which is of considerable value in several network based studies. GCAS on the other hand is a definite candidate for this purpose. Furthermore, GCN performance could suffer when information flow over longer paths are required. One way to address this would be to add additional convolutional layers in GCN to realize convolution with higher order neighbors. [24] showed that additional layers (even up to 10 layers) in GCN did not improve its performance. On the contrary, it degrades the performance due to the increase in the number of parameters and overfitting. It was shown in [24] that the performance degradation with increasing depth could be prevented if not improved by using a model variant with residual connections between hidden layers.

HANRD in its current state is by no means complete. New entity types can be incorporated into it allowing for better interpretation of newly found associations. It can also be expanded using associations from sources as OMIM, Disease Ontology [39], Orphanet Rare Disease Ontology (ORDO) [40] and GO (Gene Ontology) [41], amongst other sources. When building the nodes of HANRD, overlapping terms such as *“submucosal cleft palate”* that occurs both as a HPO node (HP:0000176) and an ORPHANET node (ORPHA:155878) were found. We dealt with such ambiguity by letting both nodes exist separately in HANRD, and connecting them with an edge weight of ’1’ to imply that they are conceptually the same. Another approach would be to merge the two nodes by choosing either of the nodes. However, this would need a consistent prioritization scheme. The edges of HANRD such as gene-gene, disease-gene and phenotype-disease represent either actual physical interactions between the entities, or simply represent the co-occurrence of the pair in a biomedical context such as a MEDLINE abstract or cellular pathway. The entities of the pairs occurring together are deemed associated by the principle of *guilt by association* [16]. Further, existing approaches usually view ontological associations as distinct from such pairwise associations [22]. HANRD includes both types of associations in the same heterogeneous network while achieving superior performance.

Curated rare disease databases such as OMIM and Orphanet have a reasonable coverage of phenotype-genotype associations. However, a significant number of such associations continue to be found only in literature, primarily due to the inherent delay involved in the manual curation of literature [42]. Approaches that can comprehensively cover all known association pairs can have a significant impact in identifying novel associations for rare disease studies. We intend to extend HANRD to include association pairs extracted from the MEDLINE corpus.

Phenotypic analysis forms only one part of the solution. Genotypic analysis in the form of variant prioritization results in a list of ranked variants and the corresponding genes [7]. Variant prioritization algorithms such as SIFT [43] and POLYPHEN [44] assess the likelihood of pathogenicity using information such as residue conservation status or the effects the change is likely to have on the protein [9]. Effectively combining the results of phenotypic and genotypic analysis can significantly improve the ability to solve clinical cases.

We used the first order approximation of the graph convolution in GCAS. It would be worthwhile to study the GCAS performance using higher order approximations, albeit with increased computational cost. Experiments in [24] however showed reduced performance for higher order approximations in comparison to the first order approximation for semi-supervised node classification, possibly due to the increase in the number of parameters. It is pertinent to note that GCAS is a domain-independent algorithm while HANRD captures the domain specific known network data. We believe that the GCAS algorithm can also find applications in other domains due to its domain-independent nature.

## Conclusions

In this study, we showed that the heterogeneous network HANRD, consisting of curated, ontological and inferred associations, helped improve causal gene identification in rare diseases. Further, the improved performance exhibited by our inferencing algorithm GCAS suggests spectral graph convolution, or graph signal processing in general, as a promising approach for biomedical network analysis.

## Additional files

Additional file 1 Rare disease clinical cases from recent publications. (PDF 139 kb)

Additional file 2 Experimental Results in tabular format. (PDF 66 kb)

## Abbreviations

AUC: Area under the curve of receiver operating characteristic
BiRW: Bi-random walk
GCAS: Graph convolution-based association scoring
GCN: Graph convolutional network
HANRD: Heterogeneous association network for rare diseases
HGNC: HUGO gene nomenclature committee
HPO: Human phenotype ontology
MAXIF: MAXimum information flow
MeSH: Medical subject headings
OMIM: Online Mendelian inheritance in man
PPI: Protein-protein interactions
RWRH: Random walk with restart on heterogeneous network
TPX: TCS Pubmed eXplorer
WES: Whole exome sequencing
WGS: Whole genome sequencing
