# Supplementary material for: Phenotype-driven gene prioritization for rare diseases using graph convolution on heterogeneous networks
Source: BMC Med Genomics. 2018 Jul 6;11:57. doi: 10.1186/s12920-018-0372-8 (PMC6035401; doi:10.1186/s12920-018-0372-8)
Supplement: Supplementary file 1 — Rare disease clinical cases from recent publications. (PDF 139 kb) [file 12920_2018_372_MOESM1_ESM.pdf]

Test Cases

Internal ID	Clinical Indication and Phenotype (HPO)	Diagnosed Disorder	Orphanet ID (Diagnosed Disorder)	Gene	HGNC Gene ID	(NM) or Locus and Genomic Variant (zygosity)	Inheritance Pattern
**Stavropoulos et al**							
TCS001	HP:0010301, HP:0000252, HP:0001263	Rubinstein-Taybi Syndrome 2	ORPHA353284 (Rubinstein-Taybi syndrome due to EP300 haploinsufficiency)	EP300	3373	NM_001429.3:c.5723dupC (p.Thr1909Asnfs*164) (het)	AD
TCS003	HP:0001263, HP:0002650, HP:0001382, HP:0002079, HP:0001999	Coffin-Siris syndrome	ORPHA1465 (Coffin-Siris syndrome)	SMARCB1	11103	NM_003073.3:c.364del (p.Glu122Asnfs*21) (het)	AD
TCS004	HP:0001263, HP:0004322, HP:0100255	Alazami Syndrome	ORPHA319671 (Microcephalic primordial dwarfism, Alazami type)	LARP7	24912	NM_016648.2:c.756_757del (p.Arg253Ile*6) (hom)	AR
TCS005	HP:0007105	KAT6B-Related Disorder	ORPHA85201 (Genitopatellar syndrome), ORPHA3047 (Blepharophimosis-intellectual disability syndrome, SBBYS type) or some other KAT6B-related disorder	KAT6B	17582	NM_012330.3:c.3021+1G>C (p?) (het)	AD
TCS006	HP:0009372, HP:0001193, HP:0007598, HP:0001762	Type C Brachydactyly	ORPHA93384 (Brachydactyly type C)	GDF5	4220	NM_000557.2:c.847G>A (p.Val283Met) (het)	AD
TCS007	HP:0001263	Neurodegeneration with brain iron accumulation-1 (NBIA1).	ORPHA216866 (Classic pantothenate kinase-associated neurodegeneration) or ORPHA157850 (Pantothenate kinase-associated neurodegeneration)	PANK2	15894	NM_153638.2:c.824_825del (p.Cys276Trpfs*15) (hom)	AR
TCS008	HP:0001263, HP:0002540, HP:0001344, HP:0003693, HP:0001266, HP:0001250, HP:0000563	Congenital disorder of deglycosylation	ORPHA137 (Congenital disorder of deglycosylation	NGLY1	17646	NM_018297.3:c.1201A>T (p.Arg401*) (hom)	AR
TCS009	HP:0000256, HP:0002126, HP:0410009, HP:0012210, HP:0003302, HP:0011342	Megalencephaly-polymicrogyria-polydactyly-hydrocephalus syndrome-1 (MPPH)	ORPHA83473 (Megalencephaly-polymicrogyria-postaxial polydactyly-hydrocephalus syndrome)	PIK3R2	8980	NM_181523.2:c.1117G>A (p.Gly373Arg) (het)	AD
TCS010	HP:0000252, HP:0001252, HP:0001250	Early Infantile Epileptic Encephalopathy 5.	ORPHA1934 (Early infantile epileptic encephalopathy)	SPTAN1	11273	NM_001130438.2:c.6947A>C (p.Gln2316Pro) (het)	AD
TCS012	HP:0001266, HP:0001263, HP:0001321, HP:0011968	Pontocerebellar Hypoplasia Type 2A.	ORPHA2524 (Pontocerebellar hypoplasia type 2)	TSEN54	27561	NM_207346.2:c.919G>T (p.Ala307Ser) (hom)	AR
TCS013	HP:0001519, HP:0011342, HP:0002194	Sotos Syndrome	ORPHA821 (Sotos syndrome)	NSD1	14234	NM_022455.4:c.3922-1G>C (p.?) (het)	AD
TCS014	HP:0012209, HP:0001642	Noonan Syndrome-like disorder with or without juvenile myelomonocytic leukemia	ORPHA363972 (Noonan syndrome-like disorder with juvenile myelomonocytic leukemia)	CBL	1541	NM_005188.3:c.1096-11_1109del (p.?) (het)	AD

TCS015	HP:0001508, HP:0001250, HP:0000256, HP:0001334, HP:0002921, HP:0002020, HP:0000545, HP:0001252, HP:0011675, HP:0000483, HP:0001647, HP:0001875	Autosomal Dominant Mental Retardation 17.	ORPHA329224 (Intellectual disability-craniofacial dysmorphism-cryptorchidism syndrome)	PACS1	30032	NM_018026.3:c.607C>T (p.Arg203Trp) (het)	AD
TCS016	HP:0001252, HP:0001263, HP:0000924	Mental retardation autosomal dominant 23	ORPHA404440 (Intellectual disability-facial dysmorphism syndrome due to SETD5 haploinsufficiency)	SETD5	25566	NM_001080517:c.1576_1580del (p.Glu526Lysfs*15) (het)	AD
TCS017	HP:0000558, HP:0000668, HP:0001999	SHORT syndrome	ORPHA3163 (SHORT syndrome)	PIK3R1	8979	NM_181523.2:c.1993G>A (p.Gly665Ser) (het)	AD
TCS018	HP:0000407, HP:0000717, HP:0001638	Autosomal Recessive hearing loss caused by GJB2 variant.	ORPHA90636 (Autosomal recessive non-syndromic sensorineural deafness type DFNB)	GJB2	4284	NM_004004.5:c.35delG (p.Gly12Valfs*2) (hom)	AR
TCS021	HP:0001107, HP:0001249, HP:0001513, HP:0007018	Oculocutaneous albinism type 1 and MC4R-linked obesity?	ORPHA352731 (Oculocutaneous albinism type 1)	TYR and MC4R	12442, 6932	NM_000372.4:c.1118C>A (p.Thr373Lys) (het)/c.1205G>A (p.Arg402Gln) (het) and NM_005912.2:c.307G>A (p.Val103Ile)	AR
TCS022	HP:0000519, HP:0001263	COL4A1-Related Disorder	ORPHA75326 (Retinal arterial tortuosity), ORPHA73229 (Autosomal dominant familial hematuria-retinal arteriolar tortuosity-contractures syndrome), ORPHA36383 (Familial vascular leukoencephalopathy), ORPHA2940 (Porencephaly) or ORPHA99810 (Familial porencephaly)	COL4A1	2202	NM_001845.4:c.2317G>A (p.Gly773Arg) (het)	AD
TCS024	HP:0002510, HP:0001263, HP:0001250, HP:0001508, HP:0000505, HP:0001537	Congenital disorder of glycosylation, type Iv and Congenital disorder of glycosylation, type IIi.	ORPHA263487 (COG5-CDG)	NGLY1 and COG5	17646, 14857	NM_001145294.1:c.517A>G p.Arg173Gly (hom) and NM_006348.3:c.1205C>T (p.Ser402Leu) (hom)	AR
TCS025	HP:0006852, HP:0002376, HP:0001945	CAPOS Syndrome and global developmental delay/Autism Spectrum disorder	ORPHA1171 (Cerebellar ataxia-areflexia-pes cavus-optic atrophy-sensorineural hearing loss syndrome)	ATP1A3 and NRXN1	801, 8008	arr 2p16.3 (51,021,507-51,358,841)x1 and NM_152296.4:c.2485G>A (p.Glu818Lys) (het)	AD
TCS026	HP:0005484, HP:0001263, HP:0002376, HP:0002123	Pontocerebellar hypoplasia, type 2E	ORPHA247198 (Progressive cerebello-cerebral atrophy)	VPS53	25608	NM_018289:c.1429C>T (p.Arg477*)/c.1716T>G (p.Ser572Arg)	AR

TCS027	HP:0009885, HP:0001087, HP:0001508, HP:0000252, HP:0030048, HP:0003072, HP:0000280, HP:0001252	Nicolaides-Baraitser syndrome	ORPHA3051 (Intellectual disability-sparse hair-brachydactyly syndrome)	SMARCA2	11098	NM_003070:c.2639C>T (p.Thr880Ile)	AD
TCS028	HP:0001250, HP:0001508, HP:0001999, HP:0000260, HP:0001382, HP:0000592, HP:0001263, HP:0001631, HP:0001943, HP:0002757, HP:0000938, HP:0001385, HP:0001903	Cutis laxa, type IIIA	ORPHA35664 (ALDH18A1-related De Barsy syndrome) or ORPHA2962 (De Barsy syndrome) or ORPHA90348 (Autosomal dominant cutis laxa)	ALDH18A1	9722	NM_002860:c.1321C>T (p.Arg441*)/c.191G>A (p.Arg64His)	AR
**Bone et al**							
TCS031	HP:0000256, HP:0000708, HP:0000718, HP:0001065, HP:0001251, HP:0001263, HP:0001290, HP:0001513, HP:0001770, HP:0001985, HP:0001999, HP:0002020, HP:0002028, HP:0002342, HP:0002360, HP:0002650, HP:0008542, HP:0011412, HP:0100512	Smith-Magenis	ORPHA819 (Smith-Magenis syndrome)	RAI1	9834	NM_030665.3:c.[2273G>A];[=]	
TCS036	HP:0000708, HP:0001250, HP:0001257, HP:0002342, HP:0007319	Combined oxidative phosphorylation deficiency 12	ORPHA314051 (Leukoencephalopathy-thalamus and brainstem anomalies-high lactate syndrome)	AARS2	21022	c.[1949G>A];	
TCS039	HP:0000518, HP:0001257, HP:0001288, HP:0002500, HP:0002518, HP:0003487, HP:0006858, HP:0006886, HP:0006937, HP:0010829, HP:0011448, HP:0011449, HP:0012534, HP:0100561	Hypomyelination with brainstem and spinal cord involvement and leg spasticity	ORPHA363412 (Hypomyelination with brain stem and spinal cord involvement and leg spasticity)	DARS	2678	NM_001349.2:c.[839A>T];[1099G>C]	
**Stelzer et al**							
TCS041	HP:0030005	Capillary leak syndrome	ORPHA188 (Systemic capillary leak syndrome)	TLN1	11845		
TCS043	HP:0002804, HP:0002803	Arthrogryposis multiplex congenita	ORPHA1037 (Arthrogryposis multiplex congenita)	MYBPC	7550		
**Lee et al**							
TCS044	HP:0002376, HP:0001250	ceroid-lipofuscinosis, neuronal 8 [MIM: 600143]	OR:168491, OR:228354, OR:79264	CLN8		p.Arg204Cys	

TCS045	HP:0001251, HP:0012444, HP:0000518, HP:0001272, HP:0002503, HP:0001263, HP:0001332, HP:0001252, HP:0002418, HP:0001270, HP:0012179, HP:0001257	autosomal recessive Joubert syndrome 3 [MIM: 608629]	OR:220493	AHI1		p.Glu1086Gly	
TCS046	HP:0007703, HP:0000662, HP:0000662, HP:0000510, HP:0000572	autosomal recessive Retinitis Pigmentosa type 39 [MIM: 613809]	OR:791	USH2A		p.Cys759Phe	
TCS047	HP:0001251, HP:0001272, HP:0001251	autosomal recessive spinocerebellar ataxia type 8 (SCAR8) [MIM: 610743]	OR:88644	SYNE1		Splice Defect	
TCS048	HP:0001274, HP:0002019, HP:0001999, HP:0000365, HP:0001609, HP:0000601, HP:0001302, HP:0000750	autosomal recessive primary microcephaly type 3 [MIM: 604804]	OR:2512	CDK5RAP2		p.Arg1481*	
TCS049	HP:0007366, HP:0001272, HP:0001347, HP:0001251, HP:0000365, HP:0001347	autosomal recessive Spastic Paraplegia type 7 [MIM: 607259]	OR:99013	SPG7		p.Gly577Ser	
TCS050	HP:0007875, HP:0000622, HP:0000551, HP:0000639, HP:0007702, HP:0007980, HP:0000510, HP:0000510	autosomal recessive cone-rod dystrophy 13 [MIM: 608194]	OR:1872	RPGRIP1		p.Asp966Ilefs	
TCS051	HP:0002804, HP:0001533, HP:0000518, HP:0001999, HP:0001328, HP:0001324, HP:0003560, HP:0002650, HP:0004322	Muscular dystrophy-dystroglycanopathy (congenital with brain and eye anomalies), type A, 14 [MIM: 615350]; Muscular dystrophy-dystroglycanopathy (congenital with mental retardation), type B, 14 [MIM: 615351]; Muscular dystrophy-dystroglycanopathy (limb-girdle), type C, 14 [MIM: 615352]	OR:363623, OR:370959, OR:370968, OR:588	GMPPB		p.Arg185Cys	
TCS052	HP:0000823, HP:0001263, HP:0001288, HP:0001347, HP:0040075, HP:0030353, HP:0004322, HP:0012506, HP:0008734	autosomal recessive Woodhouse-Sakati Syndrome [MIM: 241080]	OR:3464	DCAF17		p.Ile97Asnfs*22	
TCS053	HP:0001251, HP:0002415, HP:0009830, HP:0002500	autosomal recessive adult polyglucosan body disease [MIM: 263570]; glycogen storage disease type IV [MIM: 232500]	OR:206583, OR:308621, OR:308638, OR:308655, OR:308670, OR:308684, OR:308698, OR:308712, OR:367, OR:367	GBE1		p.Tyr329Ser	
TCS054	HP:0000717, HP:0001638, HP:0003560, HP:0003201	Duchenne muscular dystrophy (DMD) [MIM: 310200]	OR:98896	DMD		p.Gly1334*	

TCS055	HP:0002910, HP:0010526, HP:0002415, HP:0100513, HP:0001328, HP:0001257, HP:0006827, HP:0002493, HP:0002500	X-linked chronic non-spherocytic hemolytic anemia due to G6PD deficiency [MIM: 305900]		G6PD		p.Ser188Phe	
TCS056	HP:0005110, HP:0001638, HP:0001633, HP:0001634	Cardiomyopathy, dilated, 1G [MIM: 604145]; Cardiomyopathy, familial hypertrophic, 9 [MIM: 613765]	OR:154	TTN		p.Asp17434Gly, p.Cys12844*	
TCS057	HP:0001251	SYNE1-related autosomal recessive cerebellar ataxia (also known as autosomal recessive cerebellar ataxia type 1 or ARCA1) [MIM: 610743]	OR:88644	SYNE1		Splice defect, p.Lys3216*	
TCS058	HP:0001251, HP:0001272, HP:0012103	SYNE1-related autosomal recessive cerebellar ataxia (also known as autosomal recessive cerebellar ataxia type 1 or ARCA1) [MIM: 610743]	OR:88644	SYNE1		p.Arg6684*, p.Trp2646*	
TCS059	HP:0000978, HP:0001873	autosomal recessive Glanzmann thrombasthenia [MIM: 273800]	OR:849	ITGA2B		p.Ile596Thr, p.Leu147Val	
TCS060	HP:0011854, HP:0001733, HP:0012330, HP:0001977	autosomal recessive Wilson Disease [MIM:277900]	OR:905	ATP7B		p.Asn1270Ser, p.Pro984Ala	
TCS061	HP:0007754, HP:0000608, HP:0007754	autosomal recessive Stargardt Disease [MIM: 248200]	OR:827	ABCA4		p.Gly1961Glu, p.Gly863Ala	
TCS062	HP:0000023, HP:0100790	autosomal recessive 17-beta hydroxysteroid dehydrogenase III deficiency (a.k.a. male pseudohermaphroditism with gynecomastia) [MIM: 264300]	OR:46, OR:752	HSD17B3		p.Arg80Gln, p.Ser65Leu	
TCS063	HP:0004430, HP:0002721, HP:0001876	Severe combined immunodeficiency due to ADA deficiency [MIM: 102700]	OR:277	ADA		Splice defect, p.Ala329Val	
TCS064	HP:0001347, HP:0002415, HP:0009830, HP:0001257, HP:0002500	autosomal recessive spastic paraplegia type 7 [MIM: 607259]	OR:99013	SPG7		p.Ala510Val, p.Ala708Lysfs	
TCS065	HP:0003326, HP:0002099, HP:0003687, HP:0100749, HP:0012378, HP:0007185, HP:0001324, HP:0003560, HP:0003474, HP:0012531	autosomal recessive Miyoshi muscular dystrophy-1 [MIM: 254130]; autosomal recessive limb-girdle muscular dystrophy type 2B [MIM: 253601]; distal myopathy with anterior tibial onset [MIM: 606768]	OR:178400, OR:268, OR:45448	DYSF		p.Arg1693Gln, p.Ser1173*	

TCS066	HP:0003326, HP:0003326, HP:0003198	autosomal recessive limb-girdle muscular dystrophy type 2L [MIM: 611397]		ANO5		p.Asn52Ser, p.Asn64Lys*15	
TCS067	HP:0003789, HP:0001324, HP:0003560, HP:0003198	autosomal recessive congenital neuromuscular disease; central core disease [MIM: 117000]	OR:178145, OR:597, OR:598	RYR1		p.Arg1999Cys, p.Arg2241*, p.Asp708Asn	
TCS068	HP:0003202, HP:0001324, HP:0003202, HP:0003560	limb-girdle muscular dystrophy type 2B [MIM: 253601]	OR:268	DYSF		Splice defect, p.Lys1526Thr	
TCS069	HP:0002194, HP:0001252, HP:0001324, HP:0003560, HP:0003198, HP:0000508	autosomal recessive fetal akinesia deformation sequence [MIM: 208150]; familial limb-girdle myasthenia [MIM: 254300]	OR:590, OR:98913, OR:994	DOK7		p.Ser422Hisfs*34, p.Ser422Leufs*97	
TCS070	HP:0001260, HP:0002094, HP:0002875, HP:0001324, HP:0003560, HP:0003473, HP:0003198, HP:0012764	autosomal recessive familial limb-girdle myasthenia [MIM: 254300]	OR:590, OR:98913	DOK7		Splice defect, p.Ala378Serfs*30	
TCS071	HP:0001324, HP:0003560	autosomal recessive limb-girdle muscular dystrophy (LGMD) type 2A [MIM: 253600]	OR:267	CAPN3		p.Arg440Gln, p.Glu107*	
TCS072	HP:0001263, HP:0001324, HP:0003560, HP:0004322	autosomal recessive congenital muscular dystrophy (megaconial type) [MIM: 602541]	OR:280671	CHKB		p.Gln51*, p.Glu283Lys, p.Thr301Ile	
TCS073	HP:0011096, HP:0009830, HP:0003474, HP:0003401	Mitochondrial DNA depletion syndrome 4A (Alpers type) [MIM: 203700]; Mitochondrial DNA depletion syndrome 4B (MNGIE type) [MIM: 613662]; Mitochondrial recessive ataxia syndrome (includes SANDO and SCAE) [MIM: 607459]; Progressive external ophthalmoplegia, autosomal dominant [MIM: 157640]; Progressive external ophthalmoplegia, autosomal recessive [MIM: 258450]	OR:254881, OR:254886, OR:254892, OR:298, OR:70595, OR:726	POLG		p.Arg852Cys, p.Gly11Asp	
TCS074	HP:0009049, HP:0001251, HP:0002196, HP:0009830, HP:0000639, HP:0001257	autosomal recessive spastic ataxia Charlevoix-Saguenay type (ARSACS) [MIM: 270550]	OR:98	SACS		p.Arg2426*, p.Arg3875His	
TCS075	HP:0001251, HP:0001263, HP:0001250, HP:0001257, HP:0001337	autosomal recessive citrullinemia [MIM: 215700]	OR:247525	ASS1		p.Arg307Cys, see eTable 3	
TCS076	HP:0000551, HP:0000510, HP:0007754, HP:0000662, HP:0000510	late onset retinal degeneration (LORD) [MIM:605670]	OR:67042	C1QTNF5		p.Ser163Arg	

TCS077	HP:0000169, HP:0001067, HP:0001631, HP:0001647, HP:0002202, HP:0001638, HP:0001627, HP:0001635, HP:0030148, HP:0100026, HP:0011571, HP:0002202, HP:0001642, HP:0001629	Aortic valve disease [MIM: 109730]	OR:402075	NOTCH1		p.Glu794SerfsX8	
TCS078	HP:0011675, HP:0001695, HP:0001638, HP:0001699	dilated cardiomyopathy (DCM) [MIM: 115200]; hypertrophic cardiomyopathy (HCM) [MIM: 115197]	OR:300751	MYBPC3		Splice Defect	
TCS079	HP:0004749, HP:0000836, HP:0001638, HP:0012622, HP:0001635, HP:0003077, HP:0000822, HP:0002092, HP:0001645, HP:0004756	Atrial fibrillation, familial, 10 [MIM: 614022]; Brugada syndrome 1 [MIM: 601144]; Cardiomyopathy, dilated, 1E [MIM: 601154]; Heart block, nonprogressive [MIM: 113900]; Heart block, progressive, type IA [MIM: 113900]; Long QT syndrome-3 [MIM: 603830]; Sick sinus syndrome 1 [MIM: 608567]; Ventricular fibrillation, familial, 1 [MIM: 603829]	OR:101016, OR:130, OR:154, OR:166282, OR:228140, OR:334, OR:768, OR:871, OR:871	SCN5A		p.Arg523Cys	
TCS080	HP:0005110, HP:0001638, HP:0001252, HP:0011106, HP:0002202	Cardiomyopathy, dilated, 1S [MIM: 613426]; Cardiomyopathy, familial hypertrophic, 1 [MIM: 192600]; Laing distal myopathy [MIM: 160500]; Left ventricular noncompaction 5 [MIM: 613426]; Myopathy, myosin storage [MIM: 608358]; Scapuloperoneal syndrome, myopathic type [MIM: 181430]	OR:154, OR:154, OR:437572, OR:53698, OR:54260, OR:54260, OR:59135	MYH7		p.Met515Thr	
TCS081	HP:0001631, HP:0000518, HP:0000175, HP:0000568, HP:0001643	oculofaciocardiodental (OFCD) syndrome [MIM: 300166]	OR:2712, OR:568	BCOR		p.Gln1337*	
TCS082	HP:0002617, HP:0012727, HP:0012727	autosomal dominant familial thoracic aortic aneurysm type 7 [MIM: 613780]	OR:91387	MYLK		p.Asp717Tyr	
TCS083	HP:0001382, HP:0011729, HP:0003011 and HP:0000924, HP:0012531, HP:0010647	autosomal dominant Ehlers-Danlos syndrome, hypermobility type [MIM: 130020]	OR:285	TNXB		p.Asp2025Val	

TCS084	HP:0009049, HP:0002380, HP:0009027, HP:0001288, HP:0002380, HP:0001324, HP:0009830	Charcot-Marie-Tooth disease, dominant intermediate D [MIM: 607791]; Charcot-Marie-Tooth disease, type 1B [MIM: 118200]; Charcot-Marie-Tooth disease, type 2I [MIM: 607677]; Charcot-Marie-Tooth disease, type 2J [MIM: 607736]; Dejerine-Sottas disease [MIM: 145900]; Neuropathy, congenital hypomyelinating [MIM: 605253]; Roussy-Levy syndrome [MIM:180800]	OR:100046, OR:101082, OR:3115, OR:64748, OR:99942, OR:99943, OR:99951	MPZ		p.Pro151Thr	
TCS085	HP:0003234, HP:0003394, HP:0012378, HP:0001945, HP:0001427, HP:0003326, HP:0001954, HP:0002094	autosomal dominant familial periodic fever [MIM: 142680]	OR:32960	TNFRSF1A		p.Arg121Gln	
TCS086	HP:0002067, HP:0002483, HP:0001268, HP:0002307, HP:0001332, HP:0000712, HP:0000298, HP:0003470, HP:0002371, HP:0002015, HP:0000511	autosomal dominant Parkinson Disease type 11 [MIM: 607688]	OR:411602	GIGYF2		p.Ala793Val	
TCS087	HP:0010463, HP:0002617, HP:0001369, HP:0008724, HP:0010511/HP:0100807, HP:0002758, HP:0000939, HP:0000541, HP:0002650	autosomal dominant Loeys-Dietz syndrome [MIM: 610168, 610380]	OR:284973, OR:558, OR:60030, OR:91387	TGFBR2		p.Tyr470Asp	
TCS088	HP:0001430, HP:0009049, HP:0009027, HP:0003394, HP:0002487, HP:0009830	2-aminoadipic 2-oxoadipic aciduria [MIM: 204750]; Charcot-Marie-Tooth disease, axonal, type 2Q [MIM: 615025]	OR:329258, OR:79154	DHTKD1		p.Arg834*	
TCS089	HP:0000048, HP:0000047, HP:0100600, HP:0000028	46XY sex reversal type 6 [MIM: 613762]	OR:242, OR:251510	MAP3K1		p.Pro257Leu	
TCS090	HP:0002863, HP:0001903, HP:0001876	Emberger syndrome [MIM: 614038]; Immunodeficiency 21 [MIM: 614172]	OR:228423, OR:3226	GATA2		p.Asn371Lys	
TCS091	HP:0003560, HP:0003198, HP:0002380, HP:0002515, HP:0003324	Amyotrophic lateral sclerosis 14, with or without frontotemporal dementia [MIM: 613954]; Inclusion body myopathy with early-onset Paget disease and frontotemporal dementia 1 [MIM: 167320]	OR:275872, OR:52430, OR:803	VCP		p.Arg155Gly	

TCS092	HP:0007269, HP:0003324	autosomal dominant spastic paraplegia type 4 [MIM: 182601]	OR:100985	SPAST		p.Asp542Gly	
TCS093	HP:0001251, HP:0001272, HP:0000365, HP:0000726, HP:0000365, HP:0009830, HP:0001257	autosomal dominant hereditary sensory neuropathy type E1 [MIM: 614116]	OR:456318	DNMT1		p.Tyr511Cys	
TCS094	HP:0011675, HP:0001279, HP:0012668	autosomal dominant Brugada syndrome-1 [MIM: 601144]	OR:130	SCN5A		p.Ser1710Leu	
TCS095	HP:0001999, HP:0001562, HP:0000938, HP:0002652	autosomal dominant osteogenesis imperfecta (OI), type II [MIM:166210]; OI, type III [MIM: 259420]; OI, type IV [MIM: 166220]; autosomal dominant Ehlers-Danlos syndrome type VIIB [MIM: 130060]	OR:1899, OR:216804, OR:216812, OR:216820, OR:666, OR:666, OR:666, OR:99875, OR:99876	COL1A2		p.Gly1012Ser	
TCS096	HP:0001288, HP:0100021, HP:0001263, HP:0002141, HP:0002136	autosomal dominant Angelman syndrome [MIM: 105830]	OR:72	UBE3A		p.Leu825Phefs*15	
TCS097	HP:0002888, HP:0002888	autosomal dominant Li-Fraumeni Syndrome [MIM: 151623]	OR:524	TP53		p.Arg158His	
TCS098	HP:0002607, HP:0100568, HP:0012378, HP:0000131, HP:0002895, HP:0002668, HP:0001337	autosomal dominant hereditary paraganglioma type 1 [MIM: 168000]	OR:29072	SDHD		p.Pro81Leu	
TCS099	HP:0100568, HP:0002668	autosomal dominant paragangliomas [MIM: 115310]	OR:29072	SDHB		p.Arg90*	
TCS100	HP:0000589, HP:0001263, HP:0000639, HP:0001642, HP:0004322, HP:0004381, HP:0001642	autosomal dominant Noonan Syndrome 1 [MIM: 163950]	OR:648	PTPN11		p.Asn308Asp	
TCS101	HP:0001724, HP:0001647, HP:0000164, HP:0001679, HP:0001627, HP:0000975, HP:0008404	autosomal dominant pachyonychia congenita [MIM: 167200]	OR:2309	KRT16		p.Gln122Arg	

TCS102	HP:0005321, HP:0002059, HP:0000453, HP:0000028, HP:0001263, HP:0001999, HP:0002032, HP:0002937, HP:0000023, HP:0040064, HP:0000272, HP:0000308, HP:0002878	autosomal dominant mandibulofacial dysostosis, Guion-Almeida type [MIM: 610536]	OR:79113	EFTUD2		Splice Defect	
TCS103	HP:0001251, HP:0001268, HP:0000726, HP:0002415, HP:0009830, HP:0000572, HP:0002500	autosomal dominant cerebral arteriopathy with subcortical infarcts; leukoencephalopathy [MIM: 125310]	OR:136	NOTCH3		p.Arg1231Cys	
TCS104	HP:0012244, HP:0008723	46XY sex reversal 3 [MIM: 612965]; Adrenocortical insufficiency; Premature ovarian failure 7 [MIM: 612964]; Spermatogenic failure 8 [MIM: 613957]	OR:242, OR:251510, OR:399805	NR5A1		p.Tyr404Asp	
TCS105	HP:0005227, HP:0002097, HP:0002097, HP:0010566	autosomal dominant Birt-Hogg-Dube syndrome [MIM: 135150]; autosomal dominant primary spontaneous pneumothorax [MIM: 173600]	OR:122, OR:2903	FLCN		p.His429Thrfs*39	
TCS106	HP:0002313, HP:0001257	autosomal dominant spastic paraplegia 4 [MIM: 182601]	OR:100985	SPAST		p.Arg562Gln	
TCS107	HP:0007018, HP:0003761, HP:0000646, HP:0012803, HP:0001396, HP:0001263, HP:0000565, HP:0010313, HP:0001399, HP:0000722, HP:0001250, HP:0007902	autosomal dominant hyperphosphatemic familial tumoral calcinosis [MIM: 211900]	OR:306661, OR:53715	FGF23		p.Gly87Asp	
TCS108	HP:0011157, HP:0001510, HP:0001328, HP:0001250	X-linked female-restricted early infantile epileptic encephalopathy type 9 (EIEE9) or Juberg-Hellman Syndrome [MIM: 300088]	OR:101039, OR:2076	PCDH19		p.Ala153Ile	
TCS109	HP:0000365, HP:0000365, HP:0001875, HP:0002895	autosomal dominant Emberger syndrome [MIM: 614038]	OR:3226	GATA2		p.Tyr376Profs*9	
TCS110	HP:0001251, HP:0000819, HP:0000639	autosomal dominant spinocerebellar ataxia-6 [MIM:183086]; episodic ataxia type 2 [MIM:108500]; familial hemiplegic migraine-1 with progressive cerebellar ataxia [MIM:141500]	OR:569, OR:97, OR:98758	CACNA1A		p.Asp302Asn	
TCS111	HP:0009049, HP:0001288, HP:0009830	autosomal recessive Charcot-Marie-Tooth disease [MIM: 607831, 607706, 608340,; 21440]	OR:101097, OR:101097, OR:217055, OR:440, OR:90797, OR:99944	GDAP1		p.Asp21Alafs*23, p.Met116Thr, p.Pro392Ser	

TCS112	HP:0100021, HP:0002019, HP:0001263, HP:0002151, HP:0030895, HP:0001276, HP:0001252	Pitt-Hopkins Syndrome [MIM: 610954]	OR:2896	TCF4		Splice defect	
TCS113	HP:0009049, HP:0003391, HP:0003011, HP:0003202, HP:0001324, HP:0009830, HP:0007269, HP:0040083	Bethlem myopathy [MIM: 158810]; Myosclerosis, congenital [MIM: 255600]; Ullrich congenital muscular dystrophy [MIM: 254090]	OR:289380, OR:610, OR:75840	COL6A2		p.Gly699Asp	
TCS114	HP:0000787, HP:0012444, HP:0001263, HP:0000826, HP:0011157, HP:0001276, HP:0001257	Mowat-Wilson syndrome [MIM: 235730]	OR:2152	ZEB2		p.Asp978Tyr	
TCS115	HP:0001263, HP:0011157, HP:0001263, HP:0001250, HP:0003643	malignant migrating partial seizures of infancy (MMPSI)		KCNT1		p.Arg474His	
TCS116	HP:0001999, HP:0002553, HP:0000218, HP:0000750, HP:0000400, HP:0000527, HP:0000637, HP:0000750, HP:0000682	autosomal dominant Cornelia de Lange syndrome 4 [MIM: 614701]	OR:199	RAD21		p.Leu603Pro	
TCS117	HP:0011927, HP:0100249, HP:0002363, HP:0004484, HP:0000365, HP:0001999, HP:0000968, HP:0000238, HP:0011729, HP:0001270, HP:0000407, HP:0000924, HP:0000407	fibrodysplasia ossificans progressive (FOP) [MIM: 135100]	OR:337	ACVR1		p.Arg258Gly	
TCS118	HP:0001263, HP:0000388, HP:0001250, HP:0002013	early infantile epileptic encephalopathy type 11 [MIM: 613721] or benign familial infantile seizures type 3 [MIM: 607745]	OR:140927, OR:1934, OR:306	SCN2A		p.Ser987Ile	
TCS119	HP:0000365, HP:0001263, HP:0000365, HP:0012469, HP:0001250	Cognitive impairment with or without cerebellar ataxia [MIM: 614306]; Epileptic encephalopathy, early infantile, 13 [MIM: 614558]	OR:442835	SCN8A		p.Arg850Gln	
TCS120	HP:0000483, HP:0001263, HP:0001999, HP:0000316, HP:0001252, HP:0000639, HP:0001642, HP:0000026	autosomal dominant Noonan Syndrome type 3 [MIM: 609942]	OR:648	KRAS		p.Asp153Val	

TCS121	HP:0011448, HP:0012443, HP:0001272, HP:0001321, HP:0002169, HP:0005280, HP:0001263, HP:0001347, HP:0002509, HP:0008936	Dystonia 4, torsion, autosomal dominant [MIM: 128101]; Leukodystrophy, hypomyelinating, 6 [MIM: 612438]	OR:139441, OR:98805	TUBB4A		p.Val255Ile	
TCS122	HP:0030148, HP:0000238, HP:0004415, HP:0001263, HP:0001999, HP:0001250	autosomal dominant Cardiofaciocutaneous Syndrome (CFCS) [MIM: 115150]	OR:1340	MAP2K2		p.Lys61Glu	
TCS123	HP:0001263, HP:0001999, HP:0001891, HP:0001508, HP:0004322, HP:0000486, HP:0004324	autosomal dominant mental retardation type 7 [MIM:614104]	OR:178469	DYRK1A		p.Tyr104 *	
TCS124	HP:0008689, HP:0002019, HP:0001263, HP:0012572, HP:0002039, HP:0000238, HP:0001252, HP:0000023, HP:0001601, HP:0100559, HP:0100259	megalencephaly-capillary malformation-polymicrogyria syndrome (MCAP) [MIM: 602501]	OR:60040	PIK3CA		p.Gly914Arg	
TCS125	HP:0001288, HP:0007018, HP:0000717, HP:0001999, HP:0001621, HP:0001427, HP:0003560, HP:0000722	intellectual disability with language impairment; autistic features [MIM: 613670]	OR:391372	FOXP1		p.Val423His fs*37	
TCS126	HP:0003487, HP:0002415, HP:0001427, HP:0003808, HP:0002385, HP:0001257, HP:0003324	autsomal dominant Aicardi-Goutieres syndrome type 6 [MIM: 615010]; dyschromatosis symmetrica hereditaria [MIM: 127400]	OR:41, OR:51	ADAR		p.Gly1007Arg	
TCS127	HP:0001263, HP:0001999, HP:0001510, HP:0001252, HP:0000347, HP:0000568, HP:0000588, HP:0001144, HP:0000767, HP:0001510, HP:0000541, HP:0002967, HP:0004492	Wiedemann-Steiner syndrome [MIM: 605130]	OR:319182	KMT2A(MLL)		Splice defect	
TCS128	HP:0007875, HP:0007401, HP:0000639, HP:0001105, HP:0030532	Cone-rod retinal dystrophy-2 [MIM: 120970]; Leber congenital amaurosis 7 [MIM: 613829]	OR:1872, OR:65	CRX		p.Glu42Lys	
TCS129	HP:0001263, HP:0001999, HP:0011157, HP:0001385, HP:0001382, HP:0001388, HP:0001250	Turner type intellectual disability syndrome [MIM: 300706]	OR:85328	HUWE1		p.Ala1338Val	
TCS130	HP:0001263, HP:0011157, HP:0011968, HP:0002020, HP:0004305, HP:0000787, HP:0000787, HP:0001583, HP:0001250, HP:0002179, HP:0002013	Epileptic encephalopathy, early infantile, 11 [MIM: 613721]; seizures, benign familial infantile, 3 [MIM: 607745]	OR:140927, OR:1934, OR:306	SCN2A		p.Arg853Gln	

TCS131	HP:0000717, HP:0003688, HP:0001263, HP:0011157, HP:0001276, HP:0001427, HP:0001250, HP:0002179, HP:0000637	Epileptic encephalopathy, early infantile, 11 [MIM: 613721]; seizures, benign familial infantile, 3 [MIM: 607745]	OR:140927, OR:1934, OR:306	SCN2A		p.Glu1211Lys	
TCS132	HP:0000324, HP:0001263, HP:0004325, HP:0004322	X-linked dominant Cornelia de Lange Syndrome [MIM: 30882]		HDAC8		Splice defect	
TCS133	HP:0001643, HP:0012443, HP:0004484, HP:0001263, HP:0001999, HP:0001252, HP:0002637, HP:0000347, HP:0001643	autosomal dominant Beckwith-Wiedemann syndrome [MIM: 130650]; autosomal dominant Sotos syndrome 1 [MIM: 117550]	OR:116, OR:821	NSD1		p.Gln784*	
TCS134	HP:0000987, HP:0006337, HP:0010566, HP:0000365, HP:0002808, HP:0000132, HP:0002076, HP:0000826, HP:0100699, HP:0002650, HP:0000360	autosomal dominant PTEN hamartoma tumor syndrome including Bannayan-Riley-Ruvalcaba syndrome [MIM: 153480]; Cowden syndrome 1 [MIM: 158350]; macrocephaly/autism syndrome [MIM: 605309]	OR:109, OR:201, OR:210548, OR:2969, OR:65285	PTEN		p.Ser287*	
TCS135	HP:0001288, HP:0003560, HP:0003198, HP:0009830, HP:0002086, HP:0003324	autosomal dominant Charcot-Marie-Tooth disease type 2 [MIM: 601472]; distal hereditary motor neuronopathy type V [MIM: 600794]	OR:139536, OR:99938	GARS		p.Glu333Val	
TCS136	HP:0003473, HP:0000954, HP:0000598, HP:0030148, HP:0000126, HP:0001252, HP:0000579	autosomal dominant Say-Barber-Biesecker-Young-Simpson Syndrome (SBBYSS) [MIM: 603736]	OR:3047	KAT6B		p.Gln1321Argfs*20	
TCS137	HP:0000717, HP:0001999, HP:0000286, HP:0000218, HP:0001328, HP:0001166, HP:0000275, HP:0008050, HP:0002650, HP:0000098, HP:0001238, HP:0000446, HP:0000820, HP:0001780	autism type 18 [MIM: 615032]		CHD8		p.Arg773*	
TCS138	HP:0001263, HP:0001999, HP:0001276, HP:0000347, HP:0001257, HP:0001182	intellectual disability type 7 [MIM: 614104]	OR:178469	DYRK1A		p.Lys188Ile	
TCS139	HP:0001251, HP:0012443, HP:0001321, HP:0002019, HP:0001263, HP:0001310, HP:0000486, HP:0001320	Spinocerebellar ataxia 15 [MIM: 606658]; Spinocerebellar ataxia 29, congenital nonprogressive [MIM: 117360]	OR:208513, OR:98769	ITPR1		p.Ser277Ile	
TCS140	HP:0001347, HP:0001268, HP:0001276, HP:0001347, HP:0001276, HP:0001257, HP:0000572	autosomal dominant early onset dementia with or without spastic paraparesis [MIM: 607822]	OR:1020	PSEN1		p.Leu381Val	

TCS141	HP:0004322, HP:0011328, HP:0001999, HP:0000964, HP:0001627, HP:0001252, HP:0001270, HP:0002652, HP:0000750	Noonan syndrome-like disorder with loose anagen hair [MIM: 607721]	OR:2701	SHOC2		p.Ser2Gly	
TCS142	HP:0002019, HP:0100704, HP:0001263, HP:0001999, HP:0001510, HP:0001627, HP:0000047, HP:0001252, HP:0001649, HP:0000505	Mowat-Wilson syndrome [MIM: 235730]	OR:2152	ZEB2		p.Leu727Tyrfs*7	
TCS143	HP:0000717, HP:0001263, HP:0001508, HP:0008551, HP:0001250, HP:0000407, HP:0002179	Bosch-Boonstra-Schaaf optic atrophy syndrome [MIM: 615722]	OR:401777	NR2F1		p.Cys128Arg	
TCS144	HP:0100021, HP:0001263, HP:0001252, HP:0002650, HP:0001250	generalized epilepsy; paroxysmal dyskinesia [MIM: 609446]	OR:79137	KCNMA1		p.Thr352Ala	
TCS145	HP:0002020, HP:0000717, HP:0001263, HP:0004789	autosomal dominant intellectual disability type 5 [MIM: 612621]	OR:178469	SYNGAP1		p.Arg299Profs*48	
TCS146	HP:0007018, HP:0001263, HP:0001252, HP:0000750, HP:0001328, HP:0002650	autosomal dominant intellectual disability type 6 [MIM: 613970]	OR:178469	GRIN2B		p.Arg682Cys	
TCS147	HP:0002019, HP:0001263, HP:0000824, HP:0000126, HP:0001250, HP:0004322, HP:0000074	Cortical dysplasia, complex, with other brain malformations 5 [MIM: 615763]		TUBB2A		p.Gln291Pro	
TCS148	HP:0100021, HP:0100704, HP:0001263, HP:0001250	autosomal dominant early infantile epileptic encephalopathy [MIM: 61459]; autosomal dominant nocturnal frontal lobe epilepsy 5 [MIM: 615005]	OR:98784	KCNT1		p.Arg464His	
TCS149	HP:0009049, HP:0009830	Charcot-Marie-Tooth disease, dominant intermediate D [MIM: 607791]; Charcot-Marie-Tooth disease, type 1B [MIM: 118200]; Charcot-Marie-Tooth disease, type 2I [MIM: 607677]; Charcot-Marie-Tooth disease, type 2J [MIM: 607736]; Dejerine-Sottas disease [MIM: 145900]; Neuropathy, congenital hypomyelinating [MIM: 605253]; Roussy-Levy syndrome [MIM: 180800]	OR:100046, OR:101082, OR:3115, OR:64748, OR:99942, OR:99943, OR:99951	MPZ		p.Gly167Arg	

TCS150	HP:0003269, HP:0000483, HP:0001251, HP:0003429, HP:0006789, HP:0000545, HP:0000639, HP:0002352	autosomal dominant spastic paraplegia 13 [MIM: 605280]; autosomal recessive hypomyelinating leukodystrophy 4 [MIM: 612233]	OR:100994, OR:280270, OR:280288	HSPD1		p.Ala536Val	
TCS151	HP:0003234, HP:0100814, HP:0001999, HP:0001298, HP:0001252, HP:0001480, HP:0000767, HP:0001250, HP:0002079	autosomal dominant early infantile epileptic encephalopathy-7 [MIM: 613720]	OR:439218	KCNQ2		p.Gly281Trp	
TCS152	HP:0007018, HP:0000717, HP:0001263, HP:0000709	autosomal dominant nonprogressive cerebellar ataxia with intellectual disability [MIM: 614756]	OR:314647	CAMTA1		p.Tyr1077Cys	
TCS153	HP:0001263, HP:0011157, HP:0012469, HP:0012650, HP:0001250	Cortical dysplasia, complex, with other brain malformations 5 [MIM: 615763]		TUBB2A		p.Ile345Phe	
TCS154	HP:0010296, HP:0001251, HP:0001272, HP:0001263, HP:0001999, HP:0000577, HP:0000618, HP:0001250, HP:0001257, HP:0000486	autosomal dominant dystonia 12 [MIM: 128235]; autosomal dominant alternating hemiplegia of childhood 2 [MIM: 614820]	OR:2131, OR:71517	ATP1A3		p.Gly325Asp	
TCS155	HP:0000717, HP:0001263, HP:0000819, HP:0001427, HP:0003560	autosomal dominant susceptibility to autism type 18 [MIM: 615032]		CHD8		p.Arg582*	
TCS156	HP:0001007, HP:0001263, HP:0001999, HP:0002020, HP:0011471, HP:0000998, HP:0001252, HP:0000473	autosomal dominant Wiedemann-Steiner syndrome [MIM: 605130]	OR:319182	KMT2A		p.Lys1218Glufs*4	
TCS157	HP:0001263, HP:0001999, HP:0000508, HP:0004322, HP:0000750	autosomal dominant Floating-Harbor syndrome [MIM: 136140]	OR:2044	SRCAP		p.Arg2435*	
TCS158	HP:0001640, HP:0001627, HP:0002019, HP:0001263, HP:0001999, HP:0000545, HP:0000767, HP:0000508, HP:0001250, HP:0004322, HP:0002138	autosomal dominant Noonan syndrome 1 [MIM: 163950]	OR:648	PTPN11		p.Asn308Asp	
TCS159	HP:0001263, HP:0001999, HP:0002415, HP:0001250, HP:0000026, HP:0002500	autosomal dominant Nicolaides-Baraitser syndrome [MIM: 601358]	OR:3051	SMARCA2		p.His1161Arg	
TCS160	HP:0001627, HP:0001680, HP:0001263, HP:0012572, HP:0002020, HP:0001627, HP:0001252, HP:0000388, HP:0004755	autosomal dominant Kabuki syndrome [MIM: 147920]	OR:2322	KMT2D		p.Gln3759*	

TCS161	HP:0000483, HP:0001263, HP:0000565, HP:0001276, HP:0001252, HP:0002415, HP:0000545, HP:0000648, HP:0001290, HP:0002500	autosomal dominant Pitt-Hopkins syndrome [MIM: 610954]	OR:2896	TCF4		p.Gly656Argfs*55	
TCS162	HP:0001263, HP:0001999, HP:0000347, HP:0000639	autosomal dominant Nicolaides-Baraitser Syndrome [MIM: 601358]	OR:3051	SMARCA2		p.Asp534Tyr	
TCS163	HP:0001263, HP:0001999, HP:0001252, HP:0000023, HP:0002058, HP:0002928, HP:0011069, HP:0000028	autosomal dominant Glass Syndrome [MIM: 612313]	OR:251019	SATB2		p.Thr390Ile	
TCS164	HP:0001251, HP:0003560, HP:0003198	autosomal dominant limb-girdle muscular dystrophy [MIM: 159001], autosomal dominant congenital muscular dystrophy [MIM: 613205]; autosomal dominant Emery-Dreifuss muscular dystrophy 2 [MIM: 181350]	OR:157973, OR:261, OR:264, OR:98853	LMNA		Splice defect	
TCS165	HP:0100021, HP:0001263, HP:0001999, HP:0001288, HP:0002650	autosomal dominant intellectual disability 19 [MIM: 615075]	OR:404473	CTNNB1		p.Leu424Arg	
TCS166	HP:0000483, HP:0011995, HP:0001631, HP:0001647, HP:0002019, HP:0004484, HP:0000365, HP:0001263, HP:0001999, HP:0002020, HP:0000365, HP:0001601, HP:0001655	autosomal dominant CHARGE syndrome[MIM: 214800]		CHD7		p.His1734Serfs*3	
TCS167	HP:0002126, HP:0000365, HP:0001263, HP:0011157, HP:0000365, HP:0001252, HP:0000639, HP:0006879, HP:0001250	X-linked dominant intellectual disability; microcephaly with pontine; cerebellar hypoplasia (MICPCH) [MIM: 300749]	OR:163937	CASK		Splice defect	
TCS168	HP:0000175, HP:0004484, HP:0001263, HP:0001999, HP:0000842, HP:0000316, HP:0001943, HP:0001252, HP:0001511, HP:0000347, HP:0000278	autosomal dominant Birk-Barel intellectual disability dysmorphism syndrome [MIM: 612292]	OR:166108	KCNK9		p.Gly236Arg	

TCS169	HP:0001500, HP:0004484, HP:0001263, HP:0001999, HP:0000286, HP:0000286, HP:0011157, HP:0000218, HP:0003186, HP:0001388, HP:0002808, HP:0002003, HP:0000276 and HP:0000275, HP:0000368, HP:0000272, HP:0000767, HP:0000275, HP:0000767, HP:0002650, HP:0001250, HP:0001781	Sotos syndrome [MIM:117550]	OR:821	NSD1		p.Glu1184*	
TCS170	HP:0012443, HP:0001263, HP:0001347, HP:0001257, HP:0002136	autosomal dominant lissencephaly 3 [MIM: 611603]	OR:171680	TUBA1A		p.Val118Met	
TCS171	HP:0001263, HP:0001252, HP:0000508, HP:0004322	autosomal dominant Wiedemann-Steiner syndrome [MIM: 605130]	OR:319182	KMT2A		p.Ser3446Phefs*29	
TCS172	HP:0001508, HP:0001252, HP:0003128	Leigh syndrome, due to COX deficiency [MIM: 256000]	OR:255210, OR:255241, OR:506, OR:70474	SURF1		Splice Defect	
TCS173	HP:0001397, HP:0004420, HP:0003128, HP:0001942, HP:0012103, HP:0002928, HP:0002878, HP:0001714, HP:0002500	Leigh syndrome [MIM:256000]	OR:255210, OR:255241, OR:506, OR:70474	NDUFS8		p.Lys115Glu	
TCS174	HP:0007401, HP:0000551, HP:0000510, HP:0000510, HP:0007875, HP:0007401, HP:0000662, HP:0000639, HP:0000613	Leber congenital amaurosis [MIM: 614186]	OR:65	KCNJ13		p.Thr153Ile	
TCS175	HP:0012444, HP:0003688, HP:0001263, HP:0012572, HP:0001508, HP:0011968, HP:0011968, HP:0001510, HP:0040083	leukocyte adhesion deficiency II (LAD2) [MIM: 266265]	OR:2968, OR:99843	SLC35C1		p.Thr291Ile	
TCS176	HP:0000717, HP:0100543, HP:0001263, HP:0001263, HP:0001252, HP:0004305, HP:0001250, HP:0100716, HP:0002360	autosomal recessive cutis laxa, type IIIA (ARCL3A) [MIM: 219150]	OR:2962, OR:35664	ALDH18A 1		p.Arg765Gln	
TCS177	HP:0001999, HP:0001508, HP:0001252, HP:0000347, HP:0011220, HP:0002878, HP:0001250	autosomal recessive non-ketotic hyperglycinemia [MIM: 605899]	OR:407	GLDC		p.Pro581Arg	
TCS178	HP:0000365, HP:0000365	autosomal recessive Usher Syndrome type 2A [MIM: 276901]	OR:231178, OR:886	USH2A		p.Ser3276*	
TCS179	HP:0001263, HP:0000238, HP:0001250	pyridoxine-dependent epilepsy [MIM: 266100]	OR:3006	ALDH7A1		p.Glu427Gln	

TCS180	HP:0001324, HP:0003560, HP:0003198	autosomal recessive Limb-girdle muscular dystrophy type 2G [MIM: 601954]	OR:34514	TCAP		p.Ser11*	
TCS181	HP:0012179, HP:0030038, HP:0000365, HP:0001250	Bart-Pumphrey syndrome [MIM: 149200]; Deafness, autosomal dominant 3A [MIM: 601544]; Deafness, autosomal recessive 1A [MIM: 220290]; Hystrix-like ichthyosis with deafness [MIM: 602540]; Keratitis-ichthyosis-deafness syndrome [MIM: 148210]; Keratoderma, palmoplantar, with deafness [MIM: 148350]; Vohwinkel syndrome [MIM: 124500]	OR:2202, OR:2698, OR:477, OR:477, OR:494, OR:90635, OR:90636	GJB2		p.Val37Ile	
TCS182	HP:0001263, HP:0001999, HP:0011968, HP:0000126, HP:0001276, HP:0000347	autosomal recessive Crisponi syndrome (cold-induced sweating syndrome type 1) [MIM: 272430]	OR:157820	CRLF1		p.Pro239Alafs*91	
TCS183	HP:0000483, HP:0001251, HP:0000009, HP:0012444, HP:0002363, HP:0100543, HP:0000551, HP:0002283, HP:0011096, HP:0001263, HP:0003469, HP:0000648, HP:0007256, HP:0002273, HP:0001257	autosomal recessive adrenoleukodystrophy due to peroxisomal acyl-CoA oxidase deficiency [MIM: 264470]	OR:2971	ACOX1		p.Arg59Pro	
TCS184	HP:0009049, HP:0005109, HP:0001284, HP:0100021, HP:0001371, HP:0011096, HP:0002380, HP:0001324, HP:0009830, HP:0002650, HP:0002179	autosomal recessive Charcot-Marie-Tooth Disease (CMT) type 4C [MIM: 601596]	OR:99949	SH3TC2		p.Arg954*	
TCS185	HP:0012178, HP:0001945, HP:0002721, HP:0001744	autosomal recessive familial hemophagocytic lymphohistiocytosis type 2 [MIM: 603553]	OR:540	PRF1		p.Gln446Pro	
TCS186	HP:0001288, HP:0001347, HP:0007443, HP:0001427, HP:0001324, HP:0001257	autosomal recessive spastic paraplegia type 35 (SPG35) [MIM: 612319]	OR:171629	FA2H		p.Pro173Ser	
TCS187	HP:0001263, HP:0003128, HP:0001252, HP:0000816, HP:0012103, HP:0011968, HP:0002878, HP:0000488	autosomal recessive mitochondrial DNA depletion syndrome types 8A; 8B [MIM: 612075]	OR:254803, OR:255235, OR:298	RRM2B		p.Gly212_Leu213insSer	

TCS188	HP:0002586, HP:0001903, HP:0004348, HP:0100546, HP:0001263, HP:0001999, HP:0003774, HP:0001508, HP:0001627, HP:0000822, HP:0001642, HP:0001250, HP:0001297, HP:0000076	autosomal recessive combined oxidative phosphorylation deficiency type 15 [MIM: 611766]		MTFMT		p.Pro373Glnfs*19	
TCS189	HP:0001903, HP:0002908, HP:0002904, HP:0003128, HP:0001399, HP:0012103, HP:0001873, HP:0002910	autosomal recessive transient infantile liver failure [MIM: 613070]	OR:217371	TRMU		p.Leu253Pro	
TCS190	HP:0001251, HP:0000717, HP:0100021, HP:0001263, HP:0002376, HP:0001298, HP:0001252, HP:0001257	autosomal recessive infantile neuroaxonal dystrophy 1 (neurodegeneration with brain iron accumulation types 2A; 2B) [MIM: 256600] [MIM: 610217]	OR:35069, OR:35069	PLA2G6		p.Gly373Arg	
TCS191	HP:0000646, HP:0000483, HP:0001363, HP:0001263, HP:0001999, HP:0000621, HP:0011157, HP:0001252, HP:0000545, HP:0000639, HP:0000508, HP:0001250, HP:0000026	Alpha-thalassemia myelodysplasia syndrome, somatic [MIM: 300448]; Alpha-thalassemia/mental retardation syndrome [MIM: 301040]; Mental retardation-hypotonic facies syndrome, X-linked [MIM: 309580]	OR:231401, OR:73220, OR:847, OR:93970, OR:93971, OR:93972, OR:93973, OR:93974, OR:93975	ATRX		p.Pro190Ala	
TCS192		X-linked cone-rod dystrophy [MIM: 304020]	OR:1872	RPGR		p.Glu1060ArgfsX18	
TCS193	HP:0002027, HP:0000463, HP:0001263, HP:0001999, HP:0001508, HP:0001945, HP:0001276, HP:0009058, HP:0000527, HP:0030434, HP:0000520, HP:0001250, HP:0003196	Kabuki Syndrome [MIM: 300867]	OR:2322	KDM6A		p.Arg1279*	
TCS194	HP:0000717, HP:0004484, HP:0001999, HP:0000286, HP:0002463, HP:0011800	X-Linked Intellectual Disability Syndromes: Lujan-Fryns Syndrome [MIM:309520]; Opitz-Kaveggia Syndrome [MIM: 305450]	OR:776	MED12		p.Thr617Ala	
TCS195	HP:0004430, HP:0002955, HP:0002721, HP:0000999, HP:0000988, HP:0200043	X-linked recessive severe combined immunodeficiency [MIM: 300400]	OR:276	IL2RG		p.Val152Ala	
TCS196	HP:0003113, HP:0002900, HP:0002902, HP:0001254	autosomal recessive Bartter Syndrome type 3 [MIM: 607364]	OR:112, OR:93605	CLCNKB		p.Leu633*	

TCS197	HP:0001671, HP:0100021, HP:0001263, HP:0001999, HP:0001508, HP:0001627, HP:0001252, HP:0001684	X-linked recessive intellectual disability with hypotonic facies syndrome [MIM: 309580]	OR:73220, OR:93970, OR:93971, OR:93972, OR:93973, OR:93974, OR:93975	ATRX		p.Arg2131Gln	
TCS198	HP:0000717, HP:0001263, HP:0001999, HP:0001252	X-linked intellectual disability type 93 [MIM: 300659]		BRWD3		p.Leu1419Val	
TCS199	HP:0007663, HP:0007994, HP:0000510, HP:0000510, HP:0000572	glucose-6-phosphate deficiency in males [MIM: 305900]		G6PD		p.Ser188Phe	
TCS200	HP:0001284, HP:0001371, HP:0005684, HP:0001252, HP:0001511, HP:0001511, HP:0003198, HP:0200136, HP:0002098	autosomal recessive congenital myasthenic syndrome associated with facial dysmorphism; acetylcholine receptor deficiency [MIM: 608931]; autosomal recessive fetal akinesia deformation sequence [MIM: 208150]	OR:590, OR:98913, OR:994	RAPSN		p.Gln175Arg, p.Thr277Ala	
TCS201	HP:0009806, HP:0001510, HP:0003072, HP:0000843, HP:0002900, HP:0002902, HP:0012408, HP:0000103, HP:0004322	autosomal recessive Bartter syndrome, antenatal, type 1 [MIM: 601678]	OR:112, OR:93604	SLC12A1		p.Ala508Thr, p.Tyr245*	
TCS202	HP:0000717, HP:0000803, HP:0001263, HP:0001252, HP:0003560, HP:0002126	autosomal recessive congenital muscular dystrophy-dystroglycanopathy with brain; eye anomalies type A, 11 [MIM: 615181]	OR:588, OR:899	B3GALNT 2		p.Asp327Asn, p.Glu480_Trp485 dup	
TCS203	HP:0003150, HP:0001263, HP:0011157, HP:0002079, HP:0001250, HP:0001257	familial or infantile myoclonic epilepsy [MIM: 613577]		TBC1D24		p.Cys424Arg, p.Trp406Cys	
TCS204	HP:0007018, HP:0007018, HP:0000708, HP:0000510, HP:0000752, HP:0007018, HP:0000510, HP:0001250, HP:0000572	neuronal ceroid lipofuscinosis (NCL) [MIM:600143]	OR:168491, OR:228354, OR:79264	CLN8		p.Ala71Val, p.Leu188Valfs	
TCS205	HP:0011504, HP:0001133, HP:0000510	autosomal recessive juvenile onset macular dystrophy (Stargardt Disease) [MIM: 248200]	OR:827	CNGB3		p.Arg403Gln, p.Tyr398Cys	
TCS206	HP:0001251, HP:0000365, HP:0006292, HP:0000968, HP:0000365, HP:0000376, HP:0000695, HP:0004322, HP:0006288, HP:0004324	autosomal recessive hypomyelinating leukodystrophy type 8 [MIM:614381]	OR:88637	POLR3B		p.Thr663Ile, p.Val523Glu	

TCS207	HP:0011504, HP:0000551, HP:0030514, HP:0000662, HP:0001133, HP:0000510	autosomal recessive retinitis pigmentosa type 26 (RP26) [MIM: 608380]	OR:791	CERKL		p.Arg257*, p.Asp225Val	
TCS208	HP:0002650, HP:0002910, HP:0000961, HP:0001263, HP:0000363, HP:0002213, HP:0001252, HP:0012429, HP:0003560, HP:0000028, HP:0001583, HP:0002079	autosomal recessive progressive myoclonic epilepsy, type 6 [MIM: 614018]	OR:280620	GOSR2		Splice defect, p.Gly144Trp	
TCS209	HP:0002870, HP:0001385, HP:0001272, HP:0001321, HP:0002472, HP:0001263, HP:0000565, HP:0001385, HP:0001252, HP:0001427, HP:0003198, HP:0001321, HP:0025267, HP:0002515, HP:0002500	GM1-Gangliosidosis type 1 [MIM:230500]	OR:354, OR:79255	GLB1		p.Gly123Arg, p.Thr82Met	
TCS210	HP:0000565, HP:0012072, HP:0002321, HP:0001508, HP:0002315, HP:0010908, HP:0002076, HP:0002018, HP:0000246, HP:0001643, HP:0030148, HP:0004365	autosomal recessive 2-aminoadipic 2-oxoadipic aciduria [MIM:204750]	OR:79154	DHTKD1		p.Arg715Cys, p.Gly729Arg	
TCS211	HP:0001297, HP:0002381, HP:0001298, HP:0000738, HP:0001427, HP:0002180, HP:0001337, HP:0003324	autosomal recessive familial Mediterranean fever [MIM: 249100]	OR:342	MEFV		p.Ala744Ser, p.Lys695Arg	
TCS212	HP:0012115, HP:0001399	autosomal recessive Niemann-Pick disease type C1 (NPC1) [MIM: 257220]	OR:646	NPC1		p.Pro1080IlefsX18, p.Ser738*	
TCS213	HP:0001251, HP:0009830	Amyotrophic lateral sclerosis 4, juvenile [MIM: 602433]; Ataxia-ocular apraxia-2 [MIM: 606002]	OR:357043, OR:64753	SETX		Splice defect, p.Gly2036Arg	
TCS214	HP:0001709, HP:0001320, HP:0001263, HP:0012722, HP:0000545, HP:0001107, HP:0001010, HP:0001539, HP:0000609, HP:0011683	oculocutaneous albinism type IV [MIM: 606574]	OR:79435	SLC45A2, SLC45A2		p.Gly89Aspfs*24, p.Leu485Pro	
TCS215	HP:0001263, HP:0002013, HP:0001298, HP:0003128, HP:0001942	autosomal recessive acyl-CoA dehydrogenase-9 deficiency [MIM: 611126]	OR:99901	ACAD9, ACAD9		p.Phe120Serfs*9, p.Pro616Ser	
TCS216	HP:0006918, HP:0006789, HP:0001251, HP:0003128, HP:0001250, HP:0000988, HP:0001297, HP:0002401	autosomal recessive recurrent infections with encephalopathy, hepatic dysfunction; cardiovascular malformations [MIM: 613759]	OR:306550, OR:306550	FADD		p.Cys105Arg, p.Ser18*	

TCS217	HP:0012378, HP:0001324, HP:0003560, HP:0003198, HP:0012531, HP:0003201	autosomal recessive early-onset myopathy [MIM: 611705]; limb-girdle muscular dystrophy type 2J [MIM: 608807]	OR:140922, OR:289377	TTN		p.Arg32684Thrfs*47, p.Arg33084*	
TCS218	HP:0003128, HP:0001942, HP:0001427, HP:0002098	autosomal recessive lipoylation defect of the 2-ketoacid dehydrogenase complexes		LIPT1		p.Leu327*, p.Trp269*	
TCS219	HP:0002308, HP:0002020, HP:0000670, HP:0001263, HP:0001276, HP:0001250, HP:0001257	autosomal recessive Joubertâ€[?]s syndrome type 3 [MIM: 608629]	OR:220493	AHI1		p.Cys854Phe, p.Ser1123Phe	
TCS220	HP:0000083, HP:0001394, HP:0001394, HP:0001508, HP:0001399, HP:0012120	autosomal recessive methylmalonic aciduria [MIM: 251000]	OR:27, OR:289916, OR:79312	MUT		p.Ala631Glnfs*17, p.Arg108Cys	
TCS221	HP:0001638	autosomal recessive combined oxidative phosphorylation deficiency type 3 (COPD3) [MIM: 61050]		TSFM		p.Arg333Trp, p.Val119Leu	
TCS222	HP:0001263, HP:0001999, HP:0001252, HP:0003202, HP:0003560	autosomal recessive merosin-deficient muscular dystrophy [MIM: 607855]	OR:258	LAMA2		p.Arg683Serfs*21, p.Glu175*	
TCS223	HP:0002910, HP:0011157, HP:0001943, HP:0003128, HP:0001399, HP:0001324, HP:0003201, HP:0001250	autosomal recessive glutaric acidemia IIC [MIM: 231680]	OR:26791	ETFDH		p.Leu496Pro, p.Pro456Leu	
TCS224	HP:0000726, HP:0002415, HP:0001300, HP:0001337	autosomal recessive hypomyelinating leukodystrophy-7 [MIM: 607694]	OR:137639, OR:447893, OR:447896, OR:77295, OR:88637	POLR3A		p.Lys123del, p.Met852Val	
TCS225	HP:0009049, HP:0001251, HP:0009830	autosomal dominant myofibrillar myopathy type 4 [MIM: 609452]; autosomal dominant dilated cardiomyopathy type 1C with or without noncompaction of the left ventricular myocardium [MIM: 601493]	OR:154, OR:54260, OR:98912	LDB3		p.Arg268Cys	
TCS226	HP:0002647	autosomal dominant Ehlers-Danlos syndrome type IV [MIM: 130050]; autosomal dominant Ehlers-Danlos syndrome type III [MIM: 130020]	OR:285, OR:286	COL3A1		p.Gln1366*	

TCS227	HP:0011516, HP:0000551, HP:0000510, HP:0000551, HP:0030532	autosomal dominant optic atrophy type 1 (OPA1) [MIM: 165500]	OR:98673	OPA1		p.Arg711*	
TCS228	HP:0001640, HP:0001638, HP:0002566	dilated cardiomyopathy type 1FF [MIM: 613286]; type 2A [MIM: 611880]; familial hypertrophic cardiomyopathy type 7 [MIM: 613690]; familial restrictive cardiomyopathy [MIM: 115210]	OR:154, OR:154, OR:75249	TNNI3		p.Arg170Gly	
TCS229	HP:0002748, HP:0002757, HP:0000938, HP:0002652, HP:0100512	autosomal dominant osteogenesis imperfecta type II [MIM:166210], type III [MIM: 259420]; type IV [MIM:166220]	OR:216804, OR:216812, OR:216820, OR:666, OR:666, OR:666	COL1A2		p.Pro986Leu	
TCS230	HP:0002750, HP:0001510, HP:0001878, HP:0001971, HP:0002721, HP:0001744	chronic non-spherocytic hemolytic anemia (CNSHA) [MIM: 305900]		G6PD, STAT1		p.Arg274Trp, p.Ser188Phe	
TCS231	HP:0001263, HP:0001999, HP:0011157, HP:0000365, HP:0001302, HP:0001250	autosomal recessive auditory neuropathy; deafness [MIM:601071]	OR:178469, OR:284232, OR:90636, OR:98878	DYNC1H1, OTOF		p.Arg1567Leu, p.Glu1057Lys, p.Glu1700Gln, p.Lys1310dup	
TCS232	HP:0004313, HP:0002721, HP:0001891	common variable immunodeficiency type 2 [MIM: 240500]	OR:1572, OR:98879	TNFRSF1 3B		p.Ala279Thr, p.Cys104Arg	
TCS233	HP:0011516, HP:0000551, HP:0000510, HP:0000639, HP:0001133, HP:0000613	achromatopsia; cone dystrophy type 4 (COD4) [MIM: 613093]	OR:1871, OR:49382	PDE6C		p.Leu653Pro	
TCS234	HP:0003131, HP:0000097, HP:0000093, HP:0000556, HP:0003131, HP:0000110	autosomal recessive Bothnia retinal dystrophy [MIM: 607475]; autosomal recessive Retinitis punctata albescens [MIM: 136880]	OR:227796, OR:52427, OR:85128	RLBP1		p.Phe96_Phe99del	
TCS235	HP:0000510, HP:0000488	autosomal recessive retinitis pigmentosa type 38 [MIM: 613862]	OR:791	MERTK		p.Leu731Ser	
TCS236	HP:0002721, HP:0005352	autosomal recessive dyskeratosis congenita type 5 [MIM:615190]	OR:1775, OR:3322	RTEL1		p.Arg1010*, p.Ile449Thr	
TCS237	HP:0005110, HP:0002253, HP:0001997, HP:0000822, HP:0003233, HP:0000135, HP:0000821, HP:0001513, HP:0000510, HP:0010535, HP:0100512	autosomal recessive Retinitis Pigmentosa 49 (RP49) [MIM: 613756]	OR:791	CNGA1		p.Arg218*, p.Ser320Phe	
TCS238	HP:0001067	autosomal dominant neurofibromatosis type 1 (NF1) [MIM: 162200]	OR:363700, OR:636	NF1		p.Arg2616*	

TCS239	HP:0001999, HP:0000316, HP:0002885, HP:0008551	autosomal dominant desmoplastic medulloblastoma [MIM: 155255]; susceptibility to familial meningioma [MIM: 607174]	OR:251858, OR:251863, OR:251867, OR:263662, OR:616	SUFU		p.Tyr38Thrfs*58	
TCS240	HP:0005616, HP:0000646, HP:0001263, HP:0001999, HP:0011157, HP:0001250, HP:0004322, HP:0010049, HP:0003298, HP:0000076	autosomal dominant pseudohypoparathyroidism types Ia (PHP Ia) [MIM: 103580]; Ic (PHP Ic) [MIM: 612462]	OR:79443, OR:79444	GNAS		p.Gln294*	
TCS241	HP:0001263, HP:0001999, HP:0001510, HP:0001382, HP:0002079	autosomal dominant SHORT syndrome [MIM: 269880]	OR:3163	PIK3R1		p.Arg649Trp	
TCS242	HP:0000062, HP:0001328	autosomal dominant sex-limited 46,XY sex reversal 3 [MIM:612965]	OR:242, OR:251510	NR5A1		p.Glu51*	
TCS243	HP:0000365, HP:0000365, HP:0007703, HP:0000662, HP:0007703, HP:0000510, HP:0007843	autosomal dominant retinitis pigmentosa (type 37) [MIM: 611131]; enhanced S-cone syndrome [MIM: 268100]	OR:53540, OR:791	NR2E3		p.Gly56Arg	
TCS244	HP:0001631, HP:0000717, HP:0001263, HP:0001999, HP:0000577, HP:0000126, HP:0002904, HP:0000388, HP:0000488, HP:0001537	autosomal dominant Sotos Syndrome [MIM: 117550]	OR:821	NSD1		Splice defect	

Abbreviations used in this Supplementary File

HPO	Human Phenotype Ontology
HGNC	HUGO Gene Nomenclature Committee
NM	*RefSeq* Accession
AD	Autosomal Dominant
AR	Autosomal Recessive
