# Supplementary material for: Phenotype-driven gene prioritization for rare diseases using graph convolution on heterogeneous networks
Source: BMC Med Genomics. 2018 Jul 6;11:57. doi: 10.1186/s12920-018-0372-8 (PMC6035401; doi:10.1186/s12920-018-0372-8)
Supplement: Supplementary file 2 — Experimental Results in tabular format. (PDF 66 kb) [file 12920_2018_372_MOESM2_ESM.pdf]

Supplementary tables

	AUC50	AUC100	AUC300	AUC500	AUC1000	AUC
GCAS	0.14	0.19	0.28	0.34	0.41	0.67
$BiRW_{mod}$	0.14	0.18	0.24	0.27	0.31	0.62

Table 1 : *Average AUC_N with N=50, 100, 300, 500, 1000 and the full AUC value for 10-fold cross validation of GCAS and $BiRW_{mod}$*

	Top-10	Top-50	Top-100	Top-200	Top-300	Top-500	Top-1000	>Top-1000
GCAS	14.35	31.74	46.09	56.52	62.17	73.04	82.17	98.70
Phenomizer (Orphanet)	8.26	19.13	25.22	32.61	36.09	41.74	51.30	64.35
GCN	0.44	1.74	3.48	7.39	10.00	15.65	24.35	99.13

Table 2 : *Cumulative percentage of the 230 clinical cases where the causal gene(s) appeared within the Top-k of the ranked list of genes. The candidate methods are GCAS, Phenomizer(Orphanet) and GCN*

	Top-50	Top-100	Top-300	Top-500	Top-1000	>Top-1000
GCAS	13.05	20.85	38.70	48.54	65.89	93.80
$BiRW_{mod}$	13.19	18.59	29.96	33.67	41.03	98.69

Table 3 : *Cumulative percentage of all the phenotype-gene associations from the 230 clinical cases that appeared within the Top-k of the ranked gene list. The candidate methods are GCAS and $BiRW_{mod}$*

	Top-50	Top-100	Top-300	Top-500	Top-1000	>Top-1000
GCAS	8.82	14.45	30.27	39.45	57.64	92.36
$BiRW_{mod}$	7.55	9.55	16.91	19.18	26.36	98.27

Table 4 : *Cumulative percentage of all the phenotype-gene associations from the 230 clinical cases that appeared within the Top-k of the ranked gene list. The phenotype-gene associations that are already present in Orphanet are excluded from the calculation. The candidate methods are GCAS and $BiRW_{mod}$*

	Top-10	Top-50	Top-100	Top-200	Top-300	Top-500	Top-1000	>Top-1000
GCN_a	0.44	1.74	3.48	7.39	10.00	15.65	24.35	99.13
GCN_b	0.87	2.17	3.04	6.96	10.43	13.91	22.61	99.13

Table 5 : *Cumulative percentage of the 230 clinical cases where the causal gene(s) appeared within the Top-k of the ranked list of genes. The candidate methods are GCN_a and GCN_b*

	Top-10	Top-50	Top-100	Top-300	Top-500	Top-1000	>Top-1000
K=2	34	70	95	129	146	170	172
K=3	32	76	102	137	158	187	197
K=4	31	76	101	139	164	194	230
K=5	31	76	101	139	164	194	232
K=7	31	76	101	139	164	194	233
K=9	31	76	101	139	164	194	235

Table 6 : *Performance of HANRD for convolution depth parameter K = 2, 3, 4, 5, 7 and 9. For each K, the cumulative distribution of the number of causal genes appearing within the Top-k of the ranked gene lists from all the 230 clinical cases is plotted.*

Fold	AUC50	AUC100	AUC300	AUC500	AUC1000	AUC
1	0.11835	0.16832	0.25730	0.30461	0.37546	0.64298
2	0.15569	0.20712	0.30008	0.35127	0.42676	0.67526
3	0.11954	0.17038	0.26855	0.31864	0.39758	0.66086
4	0.17583	0.23229	0.33776	0.39310	0.46910	0.68455
5	0.14743	0.19640	0.29022	0.34274	0.42000	0.67799
6	0.12708	0.17329	0.25943	0.30807	0.38441	0.66291
7	0.12510	0.17056	0.26072	0.31432	0.39621	0.64165
8	0.13614	0.18567	0.28018	0.33349	0.41011	0.64074
9	0.14190	0.19011	0.28303	0.33763	0.41989	0.68536
10	0.14915	0.20179	0.30300	0.35682	0.43461	0.69623
MEAN	0.13962	0.18959	0.28403	0.33607	0.41341	0.66685
STDDEV	0.018	0.021	0.025	0.027	0.027	0.020

Table 7 : (*10-fold cross validation) fold-wise AUC values for* ***GCAS*** *with mean and standard deviation*

Fold	AUC50	AUC100	AUC300	AUC500	AUC1000	AUC
1	0.12087	0.16113	0.22525	0.25562	0.29958	0.62370
2	0.16280	0.19869	0.25257	0.27660	0.31366	0.63620
3	0.11806	0.15631	0.21656	0.24348	0.28110	0.59568
4	0.16757	0.20899	0.27444	0.30243	0.34190	0.64301

5	0.15327	0.18832	0.24380	0.26825	0.30101	0.59141
6	0.12739	0.12739	0.22214	0.24785	0.28563	0.58961
7	0.12627	0.16498	0.22772	0.25627	0.29847	0.61046
8	0.13523	0.17663	0.24767	0.28074	0.32330	0.63231
9	0.14972	0.19209	0.25375	0.27931	0.31811	0.62832
10	0.14502	0.18888	0.25463	0.28309	0.32087	0.61205
MEAN	0.14062	0.17634	0.24185	0.26936	0.30836	0.61628
STDDEV	0.018	0.024	0.018	0.018	0.019	0.019

Table 8 : (*10-fold cross validation) fold-wise* AUC_N *values for* ***BiRW****$_{mod}$* *with mean and standard deviation*
